# CircLIFR suppresses hepatocellular carcinoma progression by sponging miR-624-5p and inactivating the GSK-3β/β-catenin signaling pathway

**DOI:** 10.1038/s41419-022-04887-6

**Published:** 2022-05-17

**Authors:** Lei Yang, Wenliang Tan, Yingcheng Wei, Zhiqin Xie, Wenxin Li, Xiaowu Ma, Qingbin Wang, Huilong Li, Ziyu Zhang, Changzhen Shang, Yajin Chen

**Affiliations:** 1 Guangdong Provincial Key Laboratory of Malignant Tumor Epigenetics and Gene Regulation, Sun Yat-Sen Memorial Hospital, Sun Yat-Sen University, 510120 Guangzhou, China; 2 Department of Hepatobiliary Surgery, Sun Yat-sen Memorial Hospital, Sun Yat-sen University, 510120 Guangzhou, China; 3 Department of Hepatobiliary Surgery, Shenshan Medical Center, Memorial Hospital of Sun Yat-sen University, Shanwei, 516600 Guangdong China; 4 Department of Cardiology, The Eighth Affiliated Hospital, Sun Yat-sen University, 518000 Shenzhen, China

**Keywords:** Oncogenes, Liver cancer

## Abstract

Circular RNAs have been reported to play essential roles in the tumorigenesis and progression of various cancers. However, the biological processes and mechanisms involved in hepatocellular carcinoma (HCC) remain unclear. Initial RNA-sequencing data and qRT-PCR results in our cohort showed that hsa_circ_0072309 (also called circLIFR) was markedly downregulated in HCC tissues. Kaplan–Meier analysis indicated that higher levels of circLIFR in HCC patients correlated with favorable overall survival and recurrence-free survival rates. Both in vitro and in vivo experiments indicated that circLIFR inhibited the proliferation and invasion abilities of HCC cells. We therefore conducted related experiments to explore the mechanism of circLIFR in HCC. Fluorescence in situ hybridization results revealed that circLIFR was mainly located in the cytoplasm, and RNA immunoprecipitation assays indicated that circLIFR was significantly enriched by Ago2 protein. These results suggested that circLIFR may function as a sponge of miRNAs to regulate HCC progression. We further conducted bioinformatics prediction as well as dual-luciferase reporter assays, and the results of which showed that circLIFR could sponge miR-624-5p to stabilize glycogen synthase kinase 3β (GSK-3β) expression. Loss and gain of function experiments demonstrated that regulation of the expression of miR-624-5p or GSK-3β markedly affected HCC progression induced by circLIFR. Importantly, we also proved that circLIFR could facilitate the degradation of β-catenin and prevent its translocation to the nucleus in HCC cells. Overall, our study demonstrated that circLIFR acts as a tumor suppressor in HCC by regulating miR-624-5p and inactivating the GSK-3β/β-catenin signaling pathway.

## Introduction

Hepatocellular carcinoma (HCC) is the third most common cause of cancer-related mortality worldwide [1]. Owing to the lack of early presentation of symptoms, a large number of patients are diagnosed at a late stage and miss the opportunity for radical resection [2]. Despite improvements in surgical resection and systemic therapy, the overall survival (OS) of patients with HCC remains unsatisfactory [3]. Therefore, exploring new therapeutic targets and elucidating their molecular mechanisms will contribute to improve the OS of patients with HCC.

Circular RNAs (circRNAs) are produced by back-splicing of exons, introns, untranslated regions, or antisense genes [4]. To date, mounting evidences reveal that circRNAs are associated with the tumorigenesis of various cancers, including HCC [5, 6]. For example, circMTO1 is downregulated in HCC tumor tissues, and is associated with favorable OS of HCC patients. Its role may be to act as a sponge for miR-9-5p, thus increasing the expression of P21 and NOX4 [7, 8]. Researchers have demonstrated that circ-TCF4.85 is upregulated in patients with HCC and promotes HCC progression by targeting miR-486-5p [9]. Xu et al. discovered that circRNA-SORE promotes sorafenib resistance by inhibiting PRP19-mediated YBX1 degradation [10]. Recently, several studies have found that hsa_circ_0072309 is significantly downregulated in various cancers, including renal, gastric, and breast cancers [11–13]. However, the regulation of hsa_circ_0072309 and its potential mechanisms in HCC progression have not yet been elucidated.

Glycogen synthase kinase 3β (GSK-3β), a serine/threonine kinase, is involved in various biological functions, including cell invasion, apoptosis, proliferation, and angiogenesis [14]. β-catenin activation has been observed in ~10–50% of HCC cases [15]. As an essential component of the β-catenin destruction complex, GSK-3β phosphorylates β-catenin and triggers its degradation via the ubiquitin-proteasome pathway. A previous study demonstrated that the total protein level of GSK-3β was much lower in HCC tumor tissues than in adjacent liver tissues [16]. It has also been demonstrated that the activated form of GSK-3β (p-GSK-3β) is upregulated in 50% of HCC tissues and is associated with poorer OS [17]. Numerous circRNAs, such as hsa_circ_0003418 and hsa_circ_104348, have been demonstrated to participate in HCC progression by regulating the Wnt/β-catenin signaling pathway [18, 19]. However, the mechanisms of circRNAs and GSK-3β/β-catenin signaling pathway in HCC need to be further explored.

This study aimed to explore the role of hsa_circ_00072309 (circRNA derived from exons 2–5 of the *LIFR* gene, circLIFR) in HCC. Initially, we found that circLIFR overexpression (OE-circLIFR) inhibited the progression of HCC, while inhibition of circLIFR promoted the progression of HCC. Our results subsequently revealed that circLIFR could sponge miR-624-5p, thereby upregulating GSK-3β expression, further facilitating the degradation of β-catenin and ultimately inactivating the GSK-3β/β-catenin signaling pathway in HCC cells. In conclusion, our findings demonstrated that the circLIFR/miR-624-5p/GSK-3β axis may be a valuable therapeutic target for patients with HCC.

## Results

### circLIFR is downregulated in the HCC tissues

**Fig. 1:**
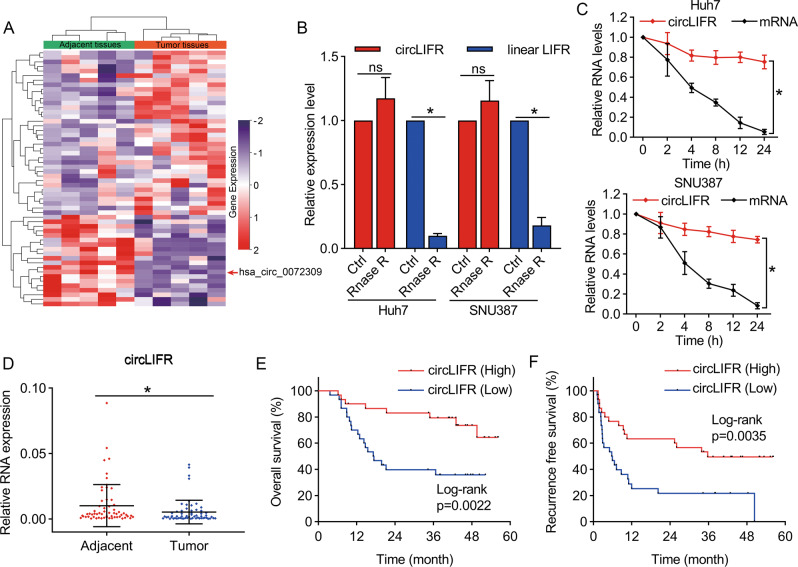
CircLIFR is downregulated in HCC and correlates with poor prognosis. **A** Heatmap of the differentially expressed circular RNAs in five HCC and adjacent non-tumor tissues. The red scales denote higher expression levels, while the purple scales represent lower expression levels. **B** qRT-PCR analysis to confirm the resistance of circLIFR and linear LIFR, with and without RNase R treatment, in Huh7 and SUN387 cells. **C** Actinomycin D treatment to assess the stability of circLIFR and LIFR mRNA over time in Huh7 and SUN387 cells. **D** Results of qRT-PCR to detect the expression of circLIFR in 60 pairs of tumor and adjacent tissues from HCC patients. **E**, **F** Kaplan–Meier analysis showing the correlation of circLIFR expression and overall survival or recurrence-free survival in HCC patients. Data represent means ± SD of three independent experiments. ^*^P < 0.05.

To identify the essential circRNAs in HCC progression, we performed high-throughput sequencing of tumors and adjacent tissues from five patients with HCC. Among the differentially expressed circRNAs (Table S2), we selected the most significantly downregulated hsa_circ_0072309 for further analysis (Fig. 1A). Initially, we searched the circBase (http://www.circbase.org/) database and found that hsa_circ_0072309 was derived from exons 2–5 of the *LIFR* gene, with a length of 580 nucleotides; therefore, we named it circLIFR. The back-splice site was identified by Sanger sequencing and was consistent with the circBase database annotation (Fig. S1A). The results in Fig. 1B revealed that the resistance of circLIFR to RNase R was greater than that of LIFR mRNA. We also treated HCC cells with actinomycin D to inhibit their transcription and found that the half-life of circLIFR was markedly greater than that of LIFR mRNA (Fig. 1C). In addition, the results of electrophoresis of PCR products verified that convergent primers could amplify linear LIFR in both complementary DNA (cDNA) and genomic DNA (gDNA), whereas divergent primers could only amplify circLIFR in cDNA (Fig. S1B). Moreover, we confirmed the expression of circLIFR in tumor and adjacent tissues from 60 pairs of patients with HCC. The results of our cohort showed that circLIFR expression was much lower in HCC tumor tissues than that in adjacent liver tissues (Fig. 1D), and higher levels of circLIFR in HCC patients correlated with favorable OS and recurrence-free survival (RFS) (Fig. 1E, F). We analyzed the correlation between circLIFR expression and clinical characteristics by *χ*^2^ test. The results demonstrated that higher expression of circLIFR was correlated with lower levels of alpha-foetoprotein (AFP), earlier Barcelona Clinic Liver Cancer (BCLC) stage, earlier tumor node metastasis (TNM) stage, smaller tumor size, and non-microvascular invasion in our cohort (Table S6). Moreover, we performed univariate and multivariate Cox regression analyses of OS and RFS in patients with HCC. The results demonstrated that TNM stage and BCLC stage were independent risk factors for OS, while no clinical characteristics in our cohort were independent risk factors for RFS (Tables S7, 8). These results demonstrated that circLIFR is downregulated in HCC and is characterized by a loop structure.

### CircLIFR inhibits proliferation of HCC cells in vitro

**Fig. 2:**
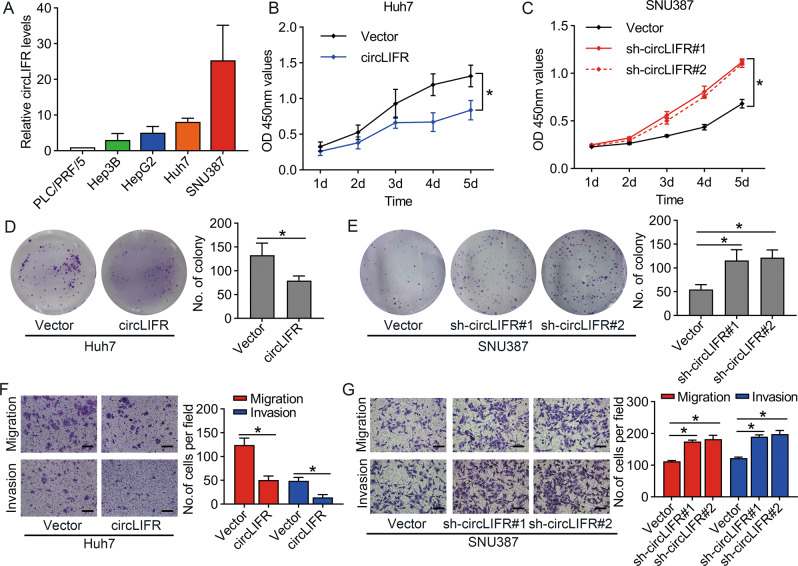
CircLIFR inhibits the progression of HCC cells in vitro. **A** qRT-PCR analysis of the circLIFR expression in five HCC cell lines. **B**, **C** Cell counting kit-8 assays to show the effect of circLIFR on cell proliferation in Huh7 and SNU387 cells. **D**, **E** Effect of circLIFR overexpression or knockdown on Huh7 and SNU387 cells examined by colony formation assay. **F**, **G** The cell migration and invasion ability, examined by Transwell assays, after overexpression of circLIFR in Huh7 cells and knockdown of circLIFR in SNU387 cells. The scale bars are 100 μm. Data represent means ± SD of three independent experiments. ^*^P < 0.05.

We first examined circLIFR RNA levels in five HCC cell lines: SNU387, HepG2, Huh7, PLC/PRF/5, and Hep3B. Because of their different origin, morphology, differentiation, grade, and tumorigenicity, the expression of circLIFR differed among these cells. The results indicated that SNU387 cells had the highest level of circLIFR expression, whereas the other four HCC cell lines had relatively low levels of circLIFR expression (Fig. 2A). Therefore, SNU387 and Huh7 cells were selected for further analysis. To investigate the biological properties of circLIFR in HCC, we downregulated circLIFR in SNU387 cells using short hairpin RNA (shRNA) and overexpressed circLIFR in Huh7 cells (Fig. S2B, C). Cell counting kit-8 (CCK-8) and colony formation assays demonstrated that overexpression of circLIFR suppressed the proliferation of Huh7 cells (Fig. 2B, D), whereas downregulation of circLIFR enhanced the proliferation ability of SNU387 cells (Fig. 2C, E). Subsequently, we also demonstrated that overexpression of circLIFR decreased the mobility of Huh7 cells (Fig. 2F), whereas inhibition of circLIFR promoted the mobility of SNU387 cells (Fig. 2G). Taken together, these findings demonstrated that circLIFR functions as a tumor suppressor in HCC cells.

### CircLIFR suppresses the growth of HCC in vivo

**Fig. 3:**
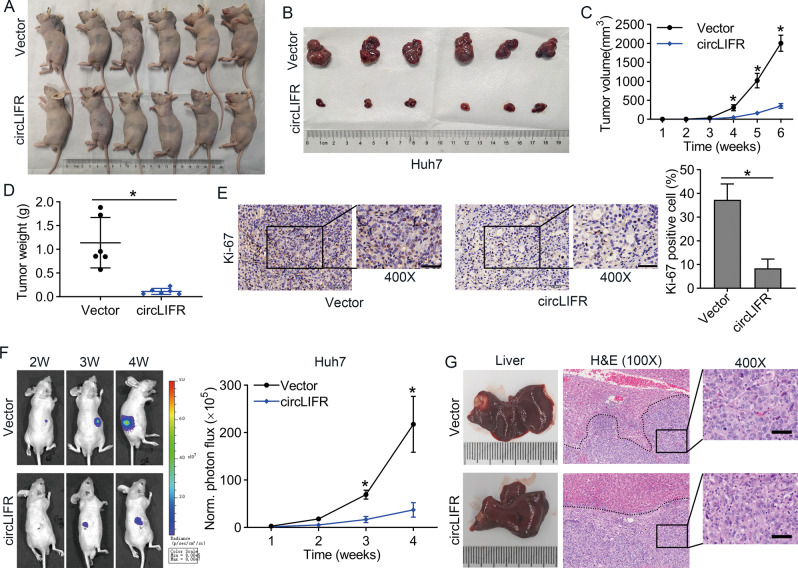
CircLIFR suppresses HCC progression in vivo. **A**, **B** Image of subcutaneous tumors in nude mice after injection of Huh7 cells (*n* = 6 for each group). **C** The tumor volume is shown to be significantly decreased in Huh7 circLIFR-overexpressing group compared with that in control group. **D** The tumor weight is significantly lower in circLIFR-overexpressing group than that in control group. **E** Representative images of xenografts stained with Ki-67 by immunohistochemistry (IHC). Images at ×200 (left panel) and ×400 (right panel) magnification. Scale bar: left = 100 μm; right = 50 μm. **F** Representative images of orthotopic xenograft tumor growth over time and quantitative analysis of the relative fluorescence intensity over time in Huh7 vector and circLIFR overexpression (OE-circLIFR) groups (*n* = 3 for each group). **G** Photographs of orthotopic xenograft tumor from Huh7 vector and OE-circLIFR groups and matched xenograft tumor hematoxylin and eosin (H&E) staining are shown. Images were photographed at ×100 (left panel) and ×400 (right panel) magnification. Scale bar: left = 200 μm; right = 50 μm. Data represent means ± SD of three independent experiments. ^*^P < 0.05.

To further evaluate the antitumor activity of circLIFR in vivo, Huh7 cells transfected with stable overexpression of circLIFR or empty vector were used to establish a subcutaneous xenograft tumor model (Fig. 3A, B). As shown in Fig. 3C, D, the tumor weight and volume in the OE-circLIFR group were much lower than those in the control group. In addition, immunohistochemical (IHC) staining results demonstrated that the ratio of Ki-67 protein-positive cells was also much lower in the OE-circLIFR group than those in the control group (Fig. 3E). In contrast, SNU387 cells transfected with circLIFR shRNA (sh-circLIFR) exhibited tumor-promoting effects in nude mice. As presented in Fig. S3A, B, downregulation of circLIFR enhanced tumor growth in mice. The tumor weight and volume in the sh-circLIFR group were much higher than those in the control group (Fig. S3C, D). IHC staining also showed that the Ki-67 levels were much higher in the sh-circLIFR group than those in the control group (Fig. S3E). The results of orthotopic xenograft tumor models showed that OE-circLIFR group exhibited lower fluorescence intensity in the liver region (Fig. 3F), whereas inhibition of circLIFR had the opposite effect (Fig. S3F). In addition, hematoxylin and eosin (H&E) staining indicated that the tumor margins in the OE-circLIFR group were smoother than those in the control group, whereas tumors in the sh-circLIFR group exhibited more aggressive margins than those in the control group (Figs. 3G, S3G).

### CircLIFR binds to miR-624-5p in HCC cells

**Fig. 4:**
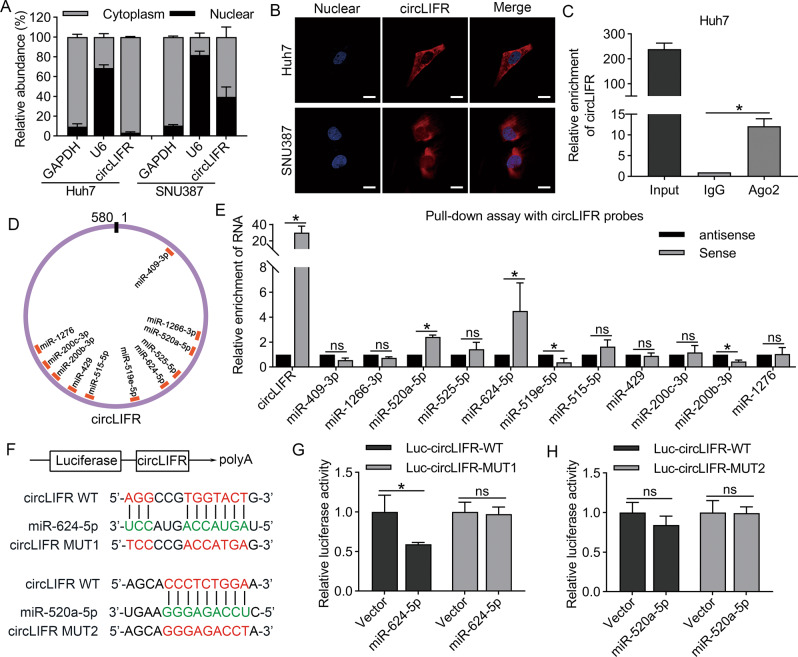
CircLIFR can bind to miR-624-5P by serving as miRNA sponges. **A** qRT-PCR assay to quantify the subcellular localization of circLIFR in Huh7 and SNU387 cells. **B** Fluorescence in situ hybridization shows circLIFR is mainly located in the cytoplasm. **C** RNA immunoprecipitation (RIP) assay demonstrating that circLIFR can sponge miRNAs by using the antibody of Ago2. **D** Schematic illustration showing the potential target miRNAs of circLIFR, predicted by miRanda and starbase. **E** RNA pull-down assay shows the miRNAs enriched in Huh7 cells. **F**–**H** The luciferase activities of circLIFR-WT, circLIFR-MUT1, and circLIFR-MUT2 plasmid luciferase reporters after co-transfection with miR-624-5p mimics or mimic NC in HCC cells. Scale bar: 10 μm. Data represent means ± SD of three independent experiments. ^*^P < 0.05.

To further explore the mechanism of circLIFR, we conducted subcellular localization detection assays which demonstrated that circLIFR was mainly located in the cytoplasm of HCC cells (Fig. 4A, B). RNA immunoprecipitation (RIP) experiments showed that circLIFR was significantly enriched by the Ago2 protein, which suggested that circLIFR could sponge miRNAs through the Ago2 protein (Figs. 4C, S4A). To investigate the downstream target miRNAs, we used two target prediction programs, miRanda (http://www.microrna.org) and starbase (http://starbase.sysu.edu.cn/), and detected 11 candidate targets in both programs (Fig. 4D). Next, we designed a biotin-circLIFR probe for RNA pull-down assays, the results of which revealed that miR-624-5p and miR-520a-5p were markedly enriched by circLIFR probe in Huh7 cells (Fig. 4E). Moreover, we further demonstrated that mimics of miR-624-5p reduced luciferase activity in Huh7 cells transfected with the circLIFR wild-type (WT) reporter, but not the circLIFR-MUT1 reporter, while miR-520a-5p mimics displayed no effect on luciferase activity of either the circLIFR-WT or circLIFR-MUT2 reporter (Fig. 4F–H). Fluorescence in situ hybridization studies revealed that circLIFR and miR-624-5p were mainly colocalized in the cytoplasm of HCC cells (Fig. S4B). In addition, we detected the expression of miR-624-5p in 60 pairs of HCC and adjacent normal tissue. The results in our cohort showed that 41 patients (68.3%) had higher miR-624-5p levels in HCC tumor tissues (Fig. S4C).

### CircLIFR inhibits progression of HCC by sponging miR-624-5p

**Fig. 5:**
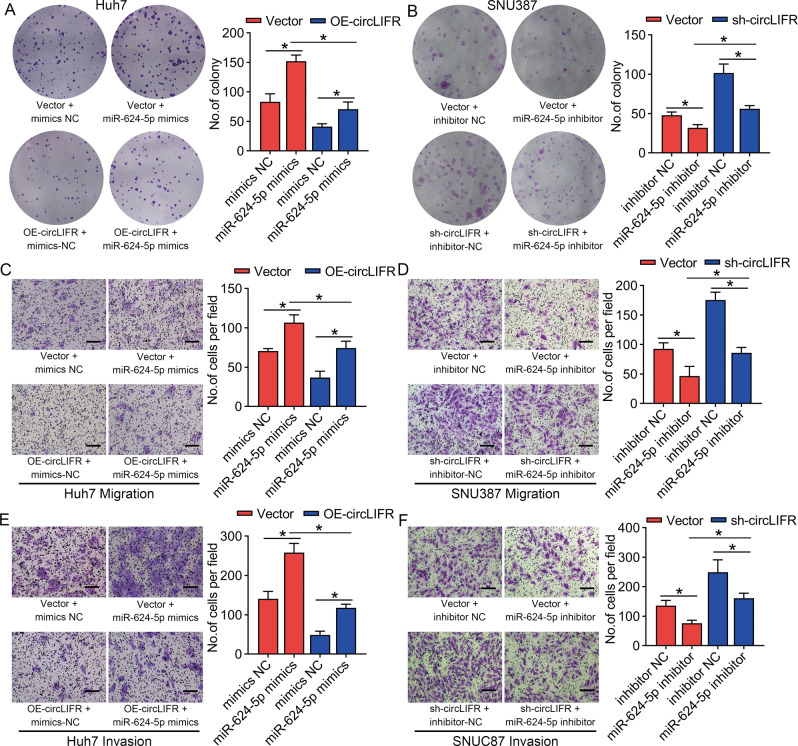
CircLIFR inhibits HCC cell proliferation, migration, and invasion via sponging miR-624-5p. **A** Colony formation assays show that transfection with miR-624-5p mimics promotes the proliferation ability of Huh7 cells and reverses the proliferation inhibition induced by overexpression of circLIFR in Huh7 cells. **B** Colony formation assays demonstrate that miR-624-5p inhibitor suppresses the proliferation ability of SNU387 cells and restores the proliferation promotion effect induced by downregulation of circLIFR in SNU387 cells. **C**–**E** Transwell assays show the effect of miR-624-5p mimics, circLIFR, or combination of both on the migration and invasion ability of Huh7 cells. **D**–**F** Transwell assays showing the effect of miR-624-5p inhibitor, circLIFR-shRNA, or combination of both on the migration and invasion ability of SNU387 cells. Scale bar: 100 μm. Data represent means ± SD of three independent experiments. ^*^P < 0.05.

To verify whether circLIFR suppressed HCC progression by sponging miR-624-5p, colony formation assays were performed. These results confirmed that circLIFR overexpression inhibited the proliferative capacity of Huh7 cells, whereas miR-624-5p mimics rescued the circLIFR overexpression-mediated inhibition of cell proliferation (Fig. 5A). In contrast, the miR-624-5p inhibitor suppressed the proliferation of SNU387 cells and restored circLIFR depletion-mediated promotion of cell proliferation (Fig. 5B). Similarly, cell migration and invasion abilities were significantly inhibited by circLIFR overexpression in Huh7 cells, while miR-624-5p mimics restored this ability to varying degrees (Fig. 5C, E). The miR-624-5p inhibitor reversed the cell mobility induced by circLIFR inhibition in SNU387 cells (Fig. 5D, F). Overall, these results suggested that circLIFR inhibits HCC progression by sponging miR-624-5p.

### GSK-3β is a downstream target of miR-624-5p

**Fig. 6:**
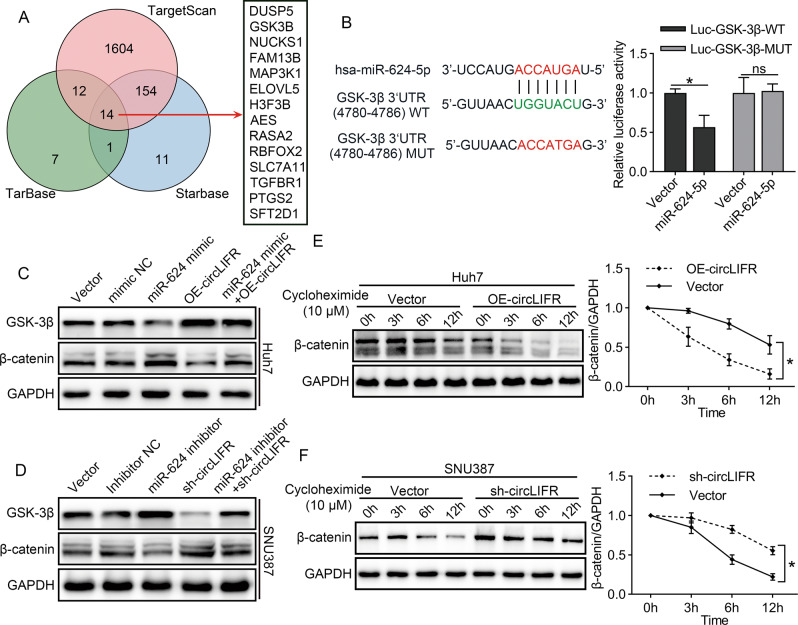
circLIFR regulates GSK-3β/β-catenin signaling pathway via sponging miR-624-5p. **A** Schematic illustration showing the potential target genes of miR-624-5p as predicted by TarBase, Starbase, and TargetScan. **B** The matched sequence of miR-624-5p with the 3′UTR of Glycogen synthase kinase 3β (GSK-3β) and luciferase reporter assays verify the relationship between miR-624-5p and GSK-3β. **C**, **D** Rescue assays demonstrate that circLIFR can modulate the expression of GSK-3β via miR-624-5p. **E**, **F** Western blot analysis for β-catenin protein levels in protein lysates, isolated at the indicated time points from HCC cells treated with 10 μM cycloheximide. Data represent means ± SD of three independent experiments. ^*^P < 0.05.

Potential targets of miR-624-5p were predicted according to TarBase, Starbase, and Targetscan [20–22]. Fourteen common genes appeared in all three prediction programs (Fig. 6A). A recent study demonstrated that miR-624-5p regulates Wnt signaling in hepatoblastoma [23]; thus, we speculated that GSK-3β might be a target of miR-624-5p. The results of dual-luciferase reporter assays further confirmed that the miR-624-5p mimic significantly inhibited the luciferase activity of the GSK-3β WT luciferase reporter plasmid, but not of the GSK-3β mutant plasmid (Fig. 6B). Moreover, we demonstrated that miR-624-5p inhibitors increased GSK-3β expression, whereas miR-624-5p mimics decreased GSK-3β expression in Huh7 and SNU387 cells (Fig. S5A).

### CircLIFR upregulates GSK-3β expression and promotes degradation of β-catenin

As GSK-3β has been reported to be a critical protein that can negatively regulate β-catenin, we investigated the regulation of the circLIFR/GSK-3β/β-catenin signaling pathway. First, we demonstrated that overexpression of circLIFR significantly upregulated GSK-3β levels in Huh7 cells, whereas knockdown of circLIFR suppressed GSK-3β levels in SNU387 cells (Fig. S5B). We then confirmed that miR-624-5p mimics significantly impaired the increase in GSK-3β levels caused by circLIFR overexpression in Huh7 cells (Fig. 6C). Similar results confirmed that the miR-624-5p inhibitor restored the effect of sh-circLIFR on GSK-3β expression in SNU387 cells (Fig. 6D). Next, we evaluated the kinetics of β-catenin degradation in HCC cells when circLIFR was either upregulated or downregulated. Our results demonstrated that the degradation rate of β-catenin was significantly higher when circLIFR was overexpressed in Huh7 cells and significantly lower when circLIFR was knocked down in SNU387 cells (Fig. 6E, F). Furthermore, we confirmed that upregulation of circLIFR expression in HCC cells prevented the nuclear translocation of β-catenin, whereas circLIFR knockdown promoted the nuclear translocation of β-catenin (Fig. S5C, D). IHC staining of subcutaneous tumors for GSK-3β and β-catenin demonstrated that overexpression of circLIFR markedly increased the expression of GSK-3β but inhibited the expression of β-catenin in Huh7 cell-derived xenograft tumors. In contrast, downregulation of circLIFR significantly inhibited the expression of GSK-3β but increased the expression of β-catenin in SNU387 cell-derived xenograft tumors (Fig. S5E, F).

### The effect of circLIFR on HCC progression depends on regulation of GSK-3β expression

**Fig. 7:**
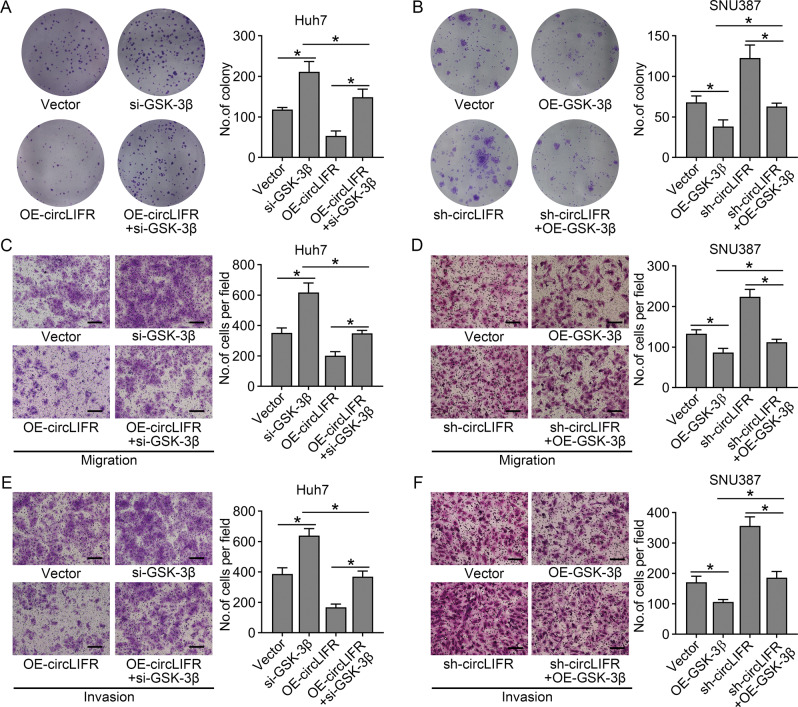
CircLIFR inhibits HCC cell progression via GSK-3β. **A**, **B** Colony formation assay showing the effect of GSK-3β and circLIFR on cell proliferation in Huh7 and SNU387 cells. **C**, **D** Transwell migration assay performed to examine the effect of GSK-3β and circLIFR on cell migration in Huh7 and SNU387 cells. **E**, **F** Transwell invasion assay performed to examine the effect of GSK-3β and circLIFR on cell invasion in Huh7 and SNU387 cells. Representative images taken at a magnification of ×100. Scale bar: 100 μm. Data represent means ± SD of three independent experiments. ^*^P < 0.05.

**Fig. 8:**
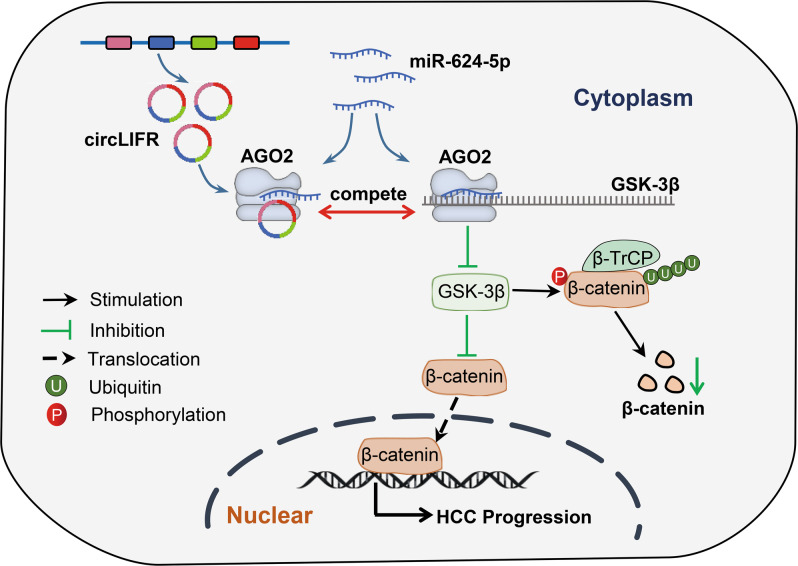
Illustrative model to show the mechanism of circLIFR in HCC. The illustrative model shows the proposed mechanism by which circLIFR upregulates GSK-3β expression, through sponging miR-624-5p, and further facilitates degradation of β-catenin and inactivates Wnt/β-catenin in HCC cells.

To verify whether circLIFR exerts tumor-suppressive effects via the circLIFR/miR-624-5p/GSK-3β axis, we performed rescue assays to assess whether GSK-3β could reverse the cellular functions of circLIFR in HCC. The in vitro experiments showed that transfection with GSK-3β short interfering RNA (si-GSK-3β) rescued the proliferation, migration, and invasion abilities of Huh7 cells inhibited by overexpression of circLIFR (Fig. 7A, C, E), whereas overexpression of GSK-3β negatively affected the cell proliferation and mobility promoted by downregulation of circLIFR in SNU387 cells (Fig. 7B, D, F). In conclusion, these results suggested that circLIFR inhibits HCC progression via the miR-624-5p/GSK-3β axis (Fig. 8).

## Discussion

Despite the improvements in systemic treatment, the OS of patients with HCC remains unsatisfactory. Therefore, exploring novel therapeutic targets and identifying their mechanisms would improve the overall prognosis of HCC. For the first time, the present study showed that circLIFR expression is markedly downregulated in HCC tumor tissues. The in vivo and in vitro experiments further verified that circLIFR could inhibit HCC progression. Moreover, we also identified that its mechanism is through regulating the miR-624-5p/GSK-3β/β-catenin axis in HCC.

Recent studies have suggested that most circRNAs are mainly located in the cytoplasm and function as sponges for miRNAs to regulate their target genes. In addition, some circRNAs are distributed in the nucleus and interact with RNA-binding proteins, thereby regulating their downstream genes [4, 24]. Our results demonstrated that circLIFR is mainly located in the cytoplasm of Huh7 and SNU387 cells, and further experiments showed that circLIFR inhibits the progression of HCC cells by sponging miR-624-5p. Similarly, Yan et al. confirmed that circLIFR is mainly located in the cytoplasm of breast cancer cells, and its mechanism involves targeting miR-492 [11]. However, a recent study demonstrated that circLIFR is predominantly located in the nucleus of bladder cancer cells, and the authors further confirmed that circLIFR could bind to MSH2 protein and act on cisplatin sensitivity [25]. Based on these contradictory results, we speculated that circLIFR exhibits a diversity of subcellular localizations in different kind of cancers.

Numerous studies have shown that many miRNAs are dysregulated in HCC [26]. Here, we confirmed the interaction between circLIFR and miR-624-5p, and rescue experiments validated that miR-624-5p mimics abrogate circLIFR-induced tumor suppression in HCC. To date, few studies have revealed the exact role of miR-624-5p in cancers. miR-624-5p is overexpressed in tumor tissues and could be a risk factor for early tumor metastasis in osteosarcoma [27]. According to another study, miR-624-5p showed much lower expression in hepatoblastoma tissues and functioned as an effective tumor suppressor [23]. These studies implied that miR-624-5p participates in different biological processes in various malignancies. The results of our investigation revealed that miR-624-5p might stimulate the progression of HCC, but its expression levels and clinical significance in HCC need to be further explored.

Activation of the β-catenin signaling pathway promotes cell proliferation and metastasis in various cancers, including HCC. Recently, it has been shown that some circRNAs regulate HCC progression by regulating the Wnt/β-catenin signaling pathway. For example, circ-DENND4C can increase TCF4 expression by competitively sponging miR-195-5p, thereby promoting nuclear translocation of β-catenin [28]. Similarly, circRNA-SORE acts as a sponge for miRNAs, thus increasing the expression of Wnt2b to promote sorafenib resistance in HCC cells [29]. The GSK-3β protein is known to negatively regulate β-catenin by phosphorylation, thus allowing its degradation by ubiquitin. A recent study showed that GSK-3β protein levels are significantly reduced in HCC, and HCC patients with low levels of GSK-3β are associated with poor OS [16]. Our study found that miR-624-5p can directly target GSK-3β and downregulate its expression, whereas circLIFR upregulates GSK-3β expression by sponging miR-624-5p. In addition, we proved that circLIFR could facilitate the degradation of β-catenin and prevent its translocation to the nucleus in HCC cells. These results demonstrated that circLIFR could upregulate GSK-3β expression and inactivate the Wnt/β-catenin signaling pathway, thereby expanding the knowledge about the molecular mechanism of HCC progression.

## Conclusions

In conclusion, our results revealed that circLIFR is markedly downregulated in HCC tumor tissues and can suppress HCC progression. Mechanistically, we demonstrated that circLIFR could upregulate GSK-3β expression by sponging miR-624-5p, thereby inhibiting HCC progression. Taken together, our findings indicated that the circLIFR/miR-624-5p/GSK-3β axis may be a meaningful target for the further clinical treatment of HCC.

## Materials and methods

### Clinical specimens and cell lines

From 2015 to 2017, surgical tissues of patients with HCC were obtained at the Sun Yat-sen Memorial Hospital of Sun Yat-Sen University (Guangzhou, China). The inclusion criteria for this study were as follows: (1) Patients were confirmed to have HCC by postoperative histopathology. (2) Patients did not receive radiofrequency ablation, chemotherapy, transcatheter arterial chemoembolisation or targeted drugs before surgery. (3) Patients without a history of other tumors. (4) Informed consent was obtained from all the patients prior to specimen collection. The exclusion criteria for this study were as follows: (1) Patients were diagnosed with cholangiocellular carcinoma or mixed hepatocellular and cholangiocellular carcinomas. (2) Patients with incomplete clinical information or follow-up data. The clinicopathological characteristics of the patients are presented in Table S5. Huh7, HepG2, and SUN387 cell lines were purchased from the Cell Bank of the Chinese Academy of Sciences (Shanghai, China). PLC/PRF/5 and Hep3B cells were obtained from ATCC (Manassas, VA, USA). The cell lines were authenticated by Short Tandem Repeat profiling and were confirmed mycoplasma-free after PCR. All cells were cultured in RPMI 1640 medium (Gibco BRL) or DMEM (Gibco BRL, USA) supplemented with 10% fetal bovine serum.

### RNA-sequencing and bioinformatic analysis

Total RNA isolated from HCC tissues using TRIzol reagent (Invitrogen, CA, USA) was assessed using Agilent 2100 Bioanalyzer pico-RNA chips (Agilent, CA, USA). During circRNA sequencing, DNase I (Epicenter) was used to remove DNA contamination. Ribosomal RNA was removed using an Epicenter Ribo-zero rRNA Removal Kit (Epicenter, USA), and RNase R digestion (Epicenter, USA) was subsequently performed to remove linear RNA. circRNA libraries were constructed with the NEBNext^®^ Ultra™ Directional RNA Library Prep Kit for Illumina^®^ (NEB, USA) following the manufacturer’s recommendations. The Illumina sequencing data were used to analyze the expression of circRNAs in tumors and adjacent tissues, using the limma package. Significantly differentially expressed circRNAs were defined as adjusted *P* < 0.05, and fold-change ≥ 2 or ≤0.5. A heatmap of the differentially expressed genes was generated using Cluster 3.0 software.

### Construction and stable transfection of circRNA plasmids

OE-circLIFR lentivirus or control lentivirus was purchased from Shanghai Genechem Co., Ltd. (Shanghai, China). Si-GSK-3β and shRNA targeting the back-splice site of circLIFR were designed and synthesized by IGE Biotechnology Ltd. (Guangzhou, China). To construct cell lines stably overexpressing circLIFR or with knocked down circLIFR, HCC cells were transfected with OE-circLIFR lentivirus or sh-circLIFR lentivirus, respectively, followed by positive selection using 2 μg/mL puromycin (Solarbio Life Sciences, Beijing, China) after 24 h. The oligonucleotide sequences are listed in Table S3.

### Nucleic acid electrophoresis

The PCR products of gDNA and cDNA were detected using 1% agarose gel electrophoresis. PCR products were separated by electrophoresis at 110 V for 20 min and detected by UV light irradiation. GL DNA Marker 2000 (AGbio, China) was used as a DNA size standard DNA marker.

### RNase R and Actinomycin D assays

Total RNA was extracted using an RNA purification kit (EZBioscience, USA) and separated into two equivalent fractions. One fraction containing 5 μg of RNA was incubated at 37 °C for 1 h in the presence of RNase R, whereas the other fraction was incubated in the absence of RNase R, as a control. Both RNA samples were then used for qRT-PCR. The actinomycin D assay was performed by addition of 2 μg/mL actinomycin D (APExBIO, Boston, USA) to Huh7 and SNU387 cells, followed by analysis of circLIFR and linear LIFR mRNA stability by qRT-PCR.

### Cell proliferation, Transwell, western blot, and qRT-PCR assays

Colony formation assay, CCK-8 assay, Transwell assay, western blotting assay, and qRT-PCR were conducted as described in our previous studies [30, 31], and also showed in the Supplementary Materials.

### Animal experiments

A total of 6 × 10^6^ Huh7 OE-circLIFR, Huh7 vector, SNU387 sh-circLIFR, or SNU387 vector cells were randomly injected into the backs of male BALB/c nude mice aged 3–5 weeks. The sample size for each group was selected based on the premise that significant differences could be detected between groups. Tumor volumes were measured weekly using digital calipers in a blinded manner. Four to six weeks later, the mice were sacrificed, and subcutaneous tumors were harvested, weighed, and fixed in formaldehyde for IHC staining. To establish an orthotopic xenograft tumor model, 2 × 10^6^ luciferase-expressing Huh7 OE-circLIFR, Huh7 vector, SNU387 sh-circLIFR, or SNU387 vector cells were randomly implanted into the right liver parenchyma of BALB/c male nude mice aged 3–5 weeks. The sample size was selected based on feasibility and cost. Bioluminescence images were examined once a week by the Xenogen IVIS Spectrum Imaging System (Xenogen, CA, USA) in a blinded manner. After 4 weeks, mouse livers were harvested for tissue sectioning. Animal experiments were approved by the Bioethics Committee of Sun Yat-Sen University.

### Fluorescence in situ hybridization (FISH)

Huh7 and SNU387 cells (1 × 10^3^) were seeded in 24-well plates, fixed with paraformaldehyde, and then incubated with 0.5% Triton X 100. The cells were then pre-hybridized using a fluorescent in situ hybridization kit (RiboBio, Guangzhou, China). Subsequently, a Cy3-labeled circLIFR probe (RiboBio, Guangzhou, China) and a FAM-labeled miR-624-5p probe (IGE Biotechnology, Guangzhou, China) were hybridized with HCC cells at 37 °C overnight. Finally, nuclei were stained with DAPI (CWBio, Beijing, China) and photographed. Probe sequences are listed in Table S3.

### Pull-down assay with biotin-labeled circLIFR probes

Sense and antisense biotin-labeled probes targeting the back-splice sites of circLIFR were synthesized by RiboBio (RiboBio, China). Pull-down assay was performed using Chromatin Isolation by RNA Purification Kit (Millipore, USA). Briefly, 1 × 10^7^ Huh7 cells were harvested for each reaction (antisense and sense) and then incubated with 3% formaldehyde. The cell precipitate was collected and lysed with lysis buffer. Subsequently, the supernatant was hybridized with sense or antisense probes in hybridization buffer for 6 h at 37 °C. Next, the probe was bound to RNA and shaken with 100 μL streptavidin magnetic beads (Millipore, USA) for 30 min at 37 °C. Finally, RNA was extracted and purified for PCR. The sense and antisense probes are provided in Table S3.

### RNA immunoprecipitation (RIP)

RIP assay was performed with a Magna RIP Kit (Millipore, USA). Briefly, 2 × 10^7^ of Huh7 cells were collected and lysed. Magnetic beads conjugated with anti-Ago2 (03-110) (Millipore, USA), or anti-IgG (03-110) (Millipore, USA) were mixed with the cell lysate supernatant before incubation overnight. Subsequently, the magnetic beads were washed five times and incubated with proteinase K at 55 °C for 30 min. Finally, isolated RNA was extracted, purified, and subjected to qRT-PCR analysis.

### Statistical analysis

Statistical analysis (presented as mean ± standard deviation) was performed using GraphPad Prism version 8.0, using Student’s *t* test and two-way analysis of variance with Bonferroni correction. Survival curves were constructed using the Kaplan–Meier method and compared using the log-rank test. Univariate and multivariate analyses were performed using Cox regression. *P* < 0.05 indicates statistically significant differences.

## Supplementary information

Supplementary Tables and Figures

Supplementary materials

Supplemental Material (Original WB)

Checklist

ARRIVE Table

## Data Availability

All data needed to evaluate the conclusions in this study are presented in the paper. Additional data related to this paper may be requested from the corresponding authors.
