# Supplementary material for: CircLIFR suppresses hepatocellular carcinoma progression by sponging miR-624-5p and inactivating the GSK-3β/β-catenin signaling pathway
Source: Cell Death Dis. 2022 May 17;13(5):464. doi: 10.1038/s41419-022-04887-6 (PMC9114368; doi:10.1038/s41419-022-04887-6)

## Table S1. Sample information of five HCC patients for circRNA sequencing.

| **Patients** | **Gender** | **Age (years)** | **BCLC stage** | **TNM stage** | **Pathology** | **HBsAg** | **Child-pugh** | **Cirrhosis** | **AFP**  **(μg/L)** | **Tumor size**  **(cm)** |
| --- | --- | --- | --- | --- | --- | --- | --- | --- | --- | --- |
| H01 | Male | 66 | C | T4N0M0, Ⅲc | Poor | **+** | A | Yes | 5739 | 13×10×7 |
| H02 | Male | 61 | C | T3aN0M0, Ⅲa | Moderate | **+** | A | No | 46744 | 13.8×11×8 |
| H03 | Male | 54 | C | T3bN0M0, Ⅲb | Poor | **+** | A | Yes | 18026 | 8×6.5×6 |
| H04 | Male | 56 | C | T3bN0M0, Ⅲb | Poor | **+** | A | Yes | 61944 | 9.5×7.5×7 |
| H05 | Male | 61 | C | T4N0M0, Ⅲc | Poor | **+** | A | No | 56870 | 12×9×8 |

## Table S2. RNAseq analysis of the significantly up- and down-regulated circRNAs in HCC tumor tissues compared with adjacent tissues.

| **circBase_ID** | **mRNA gene symbol** | **Log_2_(FC)** | **P value** | **Regulation** |
| --- | --- | --- | --- | --- |
| hsa_circ_0028587 | MED13L | 4.23919 | 0.023096 | UP |
| hsa_circ_0070039 | NUP54 | 3.834811 | 0.02925 | UP |
| hsa_circ_0007909 | ATXN1 | 3.543124 | 0.008996 | UP |
| hsa_circ_0003057 | ANKH | 3.273881 | 0.024679 | UP |
| hsa_circ_0002603 | UBR3 | 3.070821 | 0.005898 | UP |
| hsa_circ_0003340 | OGDH | 2.93421 | 0.013254 | UP |
| hsa_circ_0004535 | RP4-717I23.3 | 2.663937 | 0.024634 | UP |
| hsa_circ_0069101 | TBC1D14 | 2.585906 | 0.024615 | UP |
| hsa_circ_0004083 | PRKACB | 2.500078 | 0.019486 | UP |
| hsa_circ_0001564 | CANX | 2.48912 | 0.024256 | UP |
| hsa_circ_0004887 | AURKA | 2.488649 | 0.003557 | UP |
| hsa_circ_0002484 | ZBTB44 | 2.384267 | 0.013943 | UP |
| hsa_circ_0087391 | AGTPBP1 | 2.325991 | 0.026149 | UP |
| hsa_circ_0055945 | UXS1 | 2.316198 | 0.027986 | UP |
| hsa_circ_0004628 | SCMH1 | 2.286521 | 0.010047 | UP |
| hsa_circ_0003287 | NBAS | 2.276329 | 0.016632 | UP |
| hsa_circ_0006357 | EZH2 | 2.1368 | 0.020511 | UP |
| hsa_circ_0000130 | SNX27 | 2.095782 | 0.016901 | UP |
| hsa_circ_0008303 | PTDSS1 | 2.025997 | 0.021107 | UP |
| hsa_circ_0001617 | SNX14 | 1.778532 | 0.006399 | UP |
| hsa_circ_0000640 | SCAPER | 1.737511 | 0.02677 | UP |
| hsa_circ_0008702 | GNB1 | 1.639477 | 0.019534 | UP |
| hsa_circ_0000145 | PFDN2 | 1.564632 | 0.019196 | UP |
| hsa_circ_0000620 | AAGAB | 1.563412 | 0.025935 | UP |
| hsa_circ_0007646 | DCUN1D4 | 1.542329 | 0.02365 | UP |
| hsa_circ_0087631 | HABP4 | 1.471453 | 0.025821 | UP |
| hsa_circ_0000025 | EIF4G3 | 1.468765 | 0.002806 | UP |
| hsa_circ_0004086 | GOLIM4 | 1.417084 | 0.000876 | UP |
| hsa_circ_0000727 | ANKRD11 | 1.328908 | 0.018716 | UP |
| hsa_circ_0008967 | BTBD10 | 1.303532 | 0.006183 | UP |
| hsa_circ_0006508 | VMP1 | 1.295826 | 0.005042 | UP |
| hsa_circ_0006990 | VAPA | 1.269793 | 0.009203 | UP |
| hsa_circ_0086735 | UBAP2 | 1.173672 | 0.01986 | UP |
| hsa_circ_0003221 | PTK2 | 1.0325 | 0.006813 | UP |
| hsa_circ_0007037 | ZCCHC6 | -1.03953 | 0.026428 | Down |
| hsa_circ_0003700 | FBXO9 | -1.15313 | 0.024032 | Down |
| hsa_circ_0001360 | PHC3 | -1.19961 | 0.024404 | Down |
| hsa_circ_0001438 | LARP1B | -1.39572 | 0.026155 | Down |
| hsa_circ_0000024 | HP1BP3 | -1.43097 | 0.029507 | Down |
| hsa_circ_0008774 | GPBP1L1 | -1.6755 | 0.02903 | Down |
| hsa_circ_0002372 | ATP11C | -1.67891 | 0.026918 | Down |
| hsa_circ_0007619 | LARP1B | -1.72752 | 0.019362 | Down |
| hsa_circ_0001411 | FIP1L1 | -1.76993 | 0.016233 | Down |
| hsa_circ_0003575 | CHMP5 | -1.82948 | 0.02896 | Down |
| hsa_circ_0086376 | NFIB | -2.05269 | 0.017965 | Down |
| hsa_circ_0001445 | SMARCA5 | -2.14362 | 0.001313 | Down |
| hsa_circ_0002130 | C3 | -2.3922 | 0.006751 | Down |
| hsa_circ_0004958 | PPM1D | -2.39599 | 0.020918 | Down |
| hsa_circ_0005642 | KDM8 | -2.70788 | 0.007227 | Down |
| hsa_circ_0003400 | CRBN | -2.83349 | 0.018266 | Down |
| **hsa_circ_0072309** | **LIFR** | **-2.97031** | **0.008635** | **Down** |

FC: fold change

## Table S3. The sequence of siRNA, shRNA and probes for FISH and RIP assays.

| **Targets** | **Sequence** |
| --- | --- |
| sh-circLIFR#1 | AATTAGACTGACTGCATTGCA |
| sh-circLIFR#2 | AAATTAGACTGACTGCATT |
| si-GSK-3β | CCCAAATGTCAAACTACCAAA |
| sh-NC | GTAGCCCATGTTGTAGCAA |
| miR-624-5p mimics-F | UAGUACCAGUACCUUGUGUUCA |
| miR-624-5p mimics-R | UGAACACAAGGUACUGGUACUA |
| miR-624-5p inhibitor | UGAACACAAGGUACUGGUACUA |
| Cy3-circLIFR | UGCUGCAAAACAUCAUUAAUCUGAGGAGAA  UACUGAC |
| FAM-hsa-miR-624-5p | UGAACACAAGGUACUGGUACUA |
| Biotin-circLIFR sense | GCAGTCAGTCTAATTTTACG |
| Biotin-circLIFR antisense | CGTAAAATTAGACTGACTGC |

## Table S4. The sequence of primers in this study.

| **Primers** | **Sequence** |
| --- | --- |
| GAPDH-F | GGACCTGACCTGCCGTCTAG |
| GAPDH-R | GTAGCCCAGGATGCCCTTGA |
| U6-F | CTCGCTTCGGCAGCACA |
| U6-R | AACGCTTCACGAATTTGCGT |
| d-circLIFR-F | TCCACACCGCTCAAATGTTA |
| d-circLIFR-R | ATCCAGGATGGTCGTTTCAA |
| c-circLIFR-F | ACAAGAGCAGCGGAATGTCA |
| c-circLIFR-R | TTGGAAGGTGGTGATGAGCC |
| hsa-miR-525-5p | CAGAGGGATGCACTTTCTAAA |
| hsa-miR-409-3p | AATGTTGCTCGGTGAACCCC |
| hsa-miR-200b-3p | TAATACTGCCTGGTAATGATGA |
| hsa-miR-200c-3p | TACTGCCGGGTAATGATGGA |
| hsa-miR-429 | TAATACTGTCTGGTAAAACCGT |
| hsa-miR-519e-5p | TCTCCAAAAGGGAGCACTTTC |
| hsa-miR-624-5p | TAGTACCAGTACCTTGTGTTCA |
| hsa-miR-1266-3p | CCCTGTTCTATGCCCTGAG |

## Table S5. Clinicopathological characteristics of 60 HCC patients

| **Clinicopathological Characteristics** | **Number of cases (%)** | **Clinicopathological Characteristics** | **Number of cases (%)** |
| --- | --- | --- | --- |
| Age (years) |  | Gender |  |
| ≥ 60 | 20 (33.3%) | Male | 54 (90.0%) |
| < 60 | 40 (66.7%) | Female | 6 (10.0%) |
| Cirrhosis |  | AFP (μg/L） |  |
| Yes | 29 (48.3%) | ≥ 400 | 30 (50.0%) |
| No | 31 (51.7%) | < 400 | 30 (50.0%) |
| HBV infection |  | Tumor size |  |
| Yes | 48 (80.0%) | ≥ 5 cm | 42 (70.0%) |
| No | 12 (20.0%) | < 5 cm | 18 (30.0%) |
| Histological grade |  | TNM stage |  |
| G1 | 6 (10.0%) | Ⅰ | 12 (20.0%) |
| G2 | 26 (43.3%) | Ⅱ | 14(23.3%) |
| G3 | 28 (46.7%) | Ⅲ | 31 (51.7%) |
| BCLC stage |  | Ⅳ | 3 (5.0%) |
| A | 17 (28.3%) | MVI |  |
| B | 4(6.7%) | Yes | 39 (65.0%) |
| C | 39 (65%) | No | 21 (35.0%) |

MVI: microvascular invasion

## Table S6 Correlation between circLIFR expression and clinicopathological features in HCC tissues (n= 60, χ2-test).

| **Characteristics** | | | **Low circLIFR**  **(n = 30)** | **High circLIFR**  **(n = 30)** | **P value** |
| --- | --- | --- | --- | --- | --- |
| Age (year) | < 60 | 18 | | 22 | 0.273 |
|  | ≥ 60 | 12 | | 8 |  |
| Gender | Male | 28 | | 26 | 0.389 |
|  | Female | 2 | | 4 |  |
| AFP | < 400 | 12 | | 20 | **0.038** |
|  | ≥ 400 | 18 | | 10 |  |
| HBV infection | Yes | 22 | | 26 | 0.197 |
|  | No | 8 | | 4 |  |
| BCLC stage | A-B | 5 | | 16 | **0.003** |
|  | C | 25 | | 14 |  |
| TNM stage | I-II | 7 | | 19 | **0.002** |
|  | III-IV | 23 | | 11 |  |
| Histological grade | G1-G2 | 15 | | 17 | 0.605 |
|  | G3 | 15 | | 15 |  |
| Tumor size | ≥ 5 cm | 26 | | 16 | **0.005** |
|  | < 5 cm | 4 | | 14 |  |
| MVI | Yes | 25 | | 14 | **0.003** |
|  | No | 5 | | 16 |  |
| Cirrhosis | Yes | 12 | | 17 | 0.196 |
|  | No | 18 | | 13 |  |

MVI: microvascular invasion

## Table S7. Univariate and Multivariate analysis various prognostic characteristics for overall survival (OS) in patients with HCC.

|  | **Univariate analysis (OS)** | | | **Multivariate analysis (OS)** | | |
| --- | --- | --- | --- | --- | --- | --- |
|  | **P** | **HR** | **95% CI** | **P** | **HR** | **95% CI** |
| Age | 0.229 | 1.602 | 0.743-3.455 |  |  |  |
| Gender | 0.794 | 0.851 | 0.255-2.840 |  |  |  |
| Tumor size | 0.008 | 5.100 | 1.531-16.989 | 0.376 | 0.495 | 0.104-2.353 |
| HBV infection | 0.015 | 0.366 | 0.164-0.820 | 0.121 | 0.499 | 0.207-1.203 |
| Histological grade | 0.343 | 1.442 | 0.677-3.074 |  |  |  |
| TNM stage | 0.001 | 5.506 | 2.066-14.669 | **0.004** | **3.874** | **1.524-9.746** |
| BCLC stage | 0.001 | 10.459 | 2.468-44.318 | **0.026** | **24.124** | **1.469-396.071** |
| AFP | 0.012 | 2.736 | 1.247-6.003 | 0.740 | 1.170 | 0.463-2.960 |
| Cirrhosis | 0.032 | 0.414 | 0.185-0.926 | 0.461 | 0.708 | 0.283-1.773 |
| circLIFR expression | 0.004 | 0.291 | 0.126-0.672 | 0.657 | 1.265 | 0.448-3.572 |
| MVI | 0.002 | 6.507 | 1.952-21.688 | 0.062 | 0.023 | 0.000-1.212 |

HR: Hazard Ratio, CI: Confidence Interval, MVI: microvascular invasion

## Table S8. Univariate and Multivariate analysis various prognostic characteristics for RFS (recurrence-free survival) in patients with HCC.

|  | **Univariate analysis (RFS)** | | | **Multivariate analysis (RFS)** | | |
| --- | --- | --- | --- | --- | --- | --- |
|  | **P** | **HR** | **95% CI** | **P** | **HR** | **95% CI** |
| Age | 0.599 | 1.192 | 0.619-2.295 |  |  |  |
| Gender | 0.714 | 0.824 | 0.292-2.324 |  |  |  |
| Tumor size | 0.005 | 3.285 | 1.440-7.490 | 0.525 | 1.442 | 0.467-4.454 |
| HBV infection | 0.501 | 0.764 | 0.350-1.671 |  |  |  |
| Histological grade | 0.722 | 1.091 | 0.676-1.761 |  |  |  |
| TNM stage | 0.001 | 1.955 | 1.326-2.883 | 0.111 | 1.546 | 0.905-2.644 |
| BCLC stage | 0.014 | 1.604 | 1.102-2.335 | 0.506 | 1.760 | 0.333-9.290 |
| AFP | 0.035 | 1.982 | 1.051-3.736 | 0.476 | 1.288 | 0.643-2.581 |
| Cirrhosis | 0.208 | 0.667 | 0.354-1.253 |  |  |  |
| Child-Pugh | 0.526 | 1.912 | 0.258-14.144 |  |  |  |
| circLIFR expression | 0.005 | 0.388 | 0.201-0.749 | 0.284 | 0.633 | 0.275-1.461 |
| MVI | 0.026 | 2.228 | 1.102-4.503 | 0.601 | 0.470 | 0.028-7.983 |

HR: Hazard Ratio, CI: Confidence Interval, MVI: microvascular invasion

Supplementary Figure 1


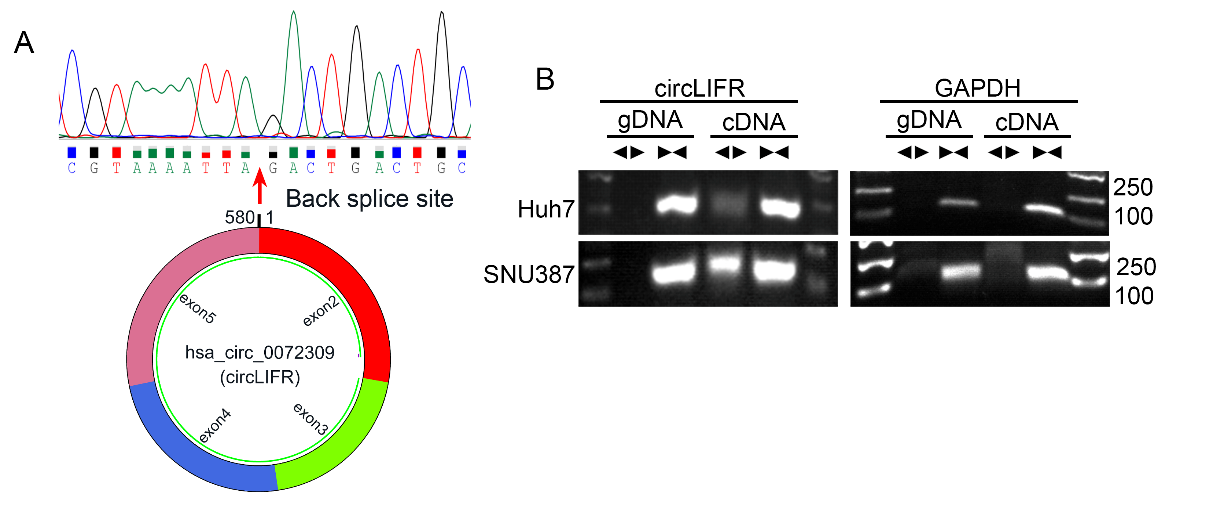


Supplementary Figure 2


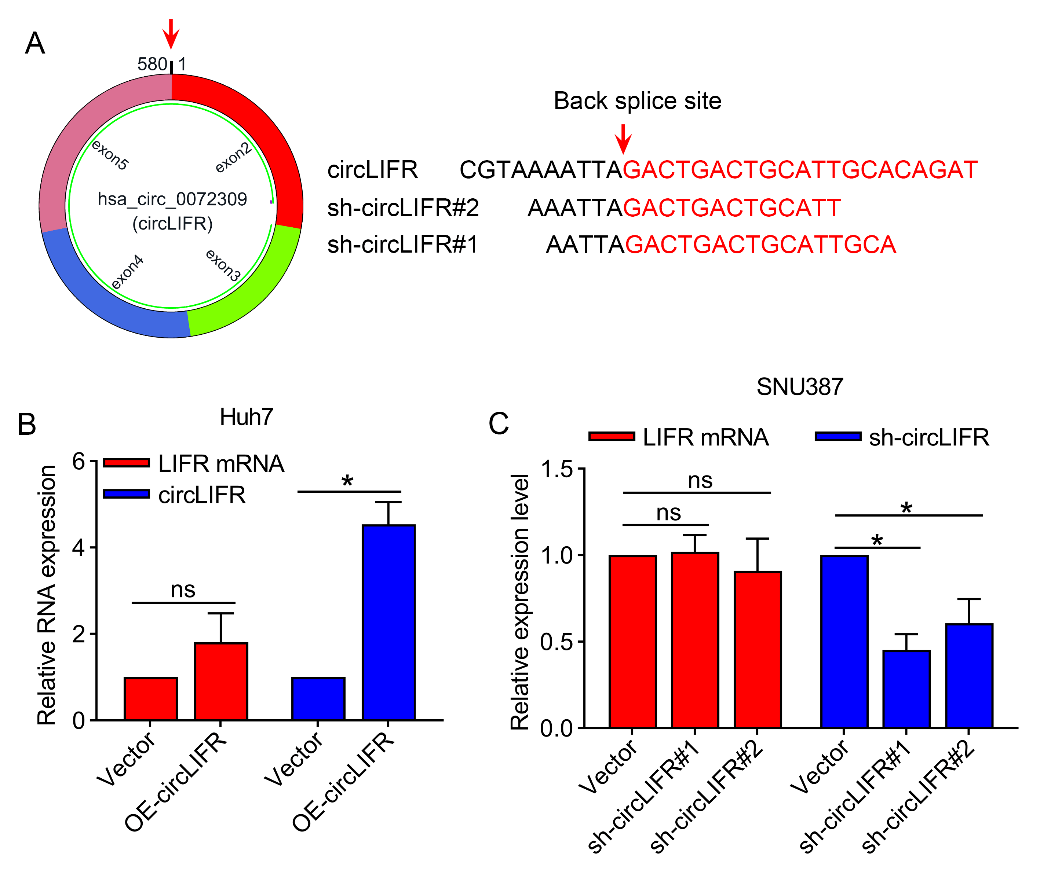


Supplementary Figure 3


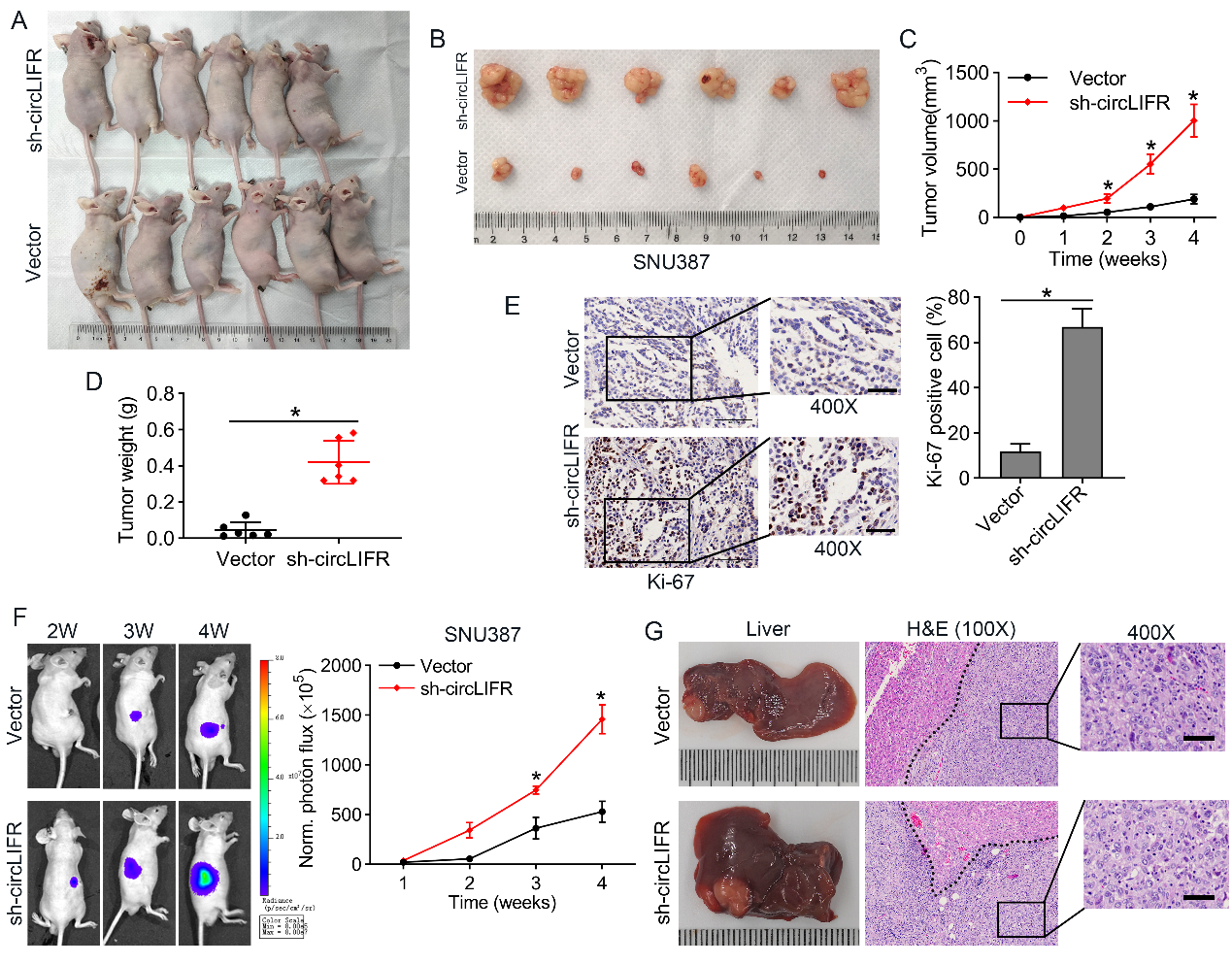


Supplementary Figure 4


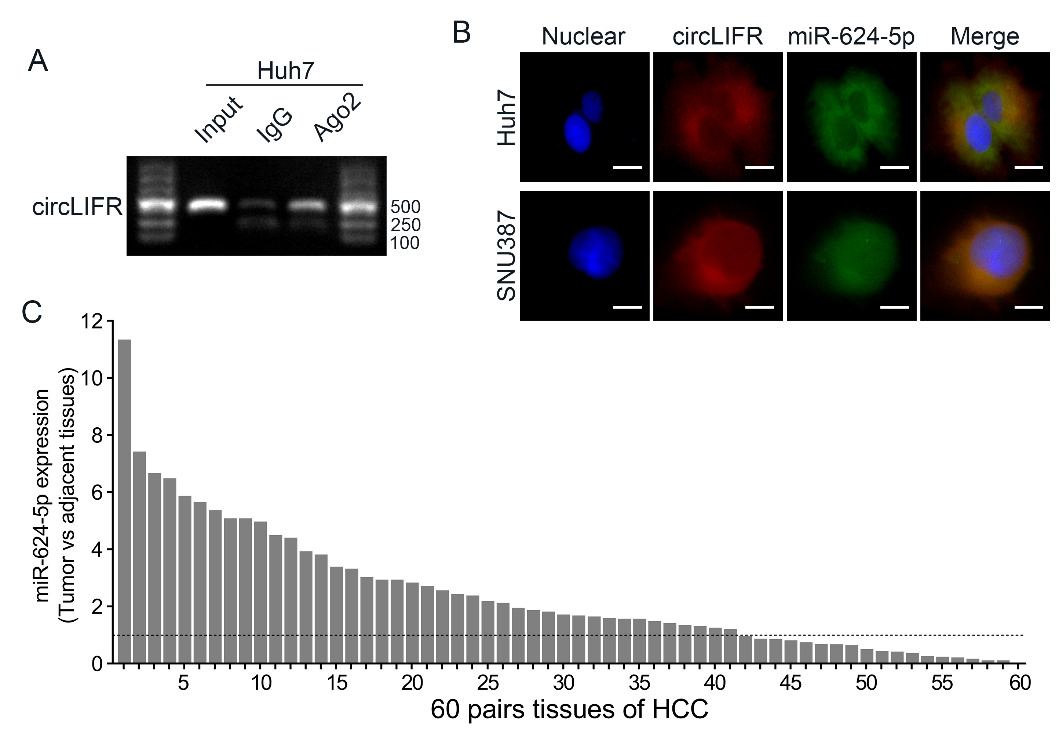


Supplementary Figure 5


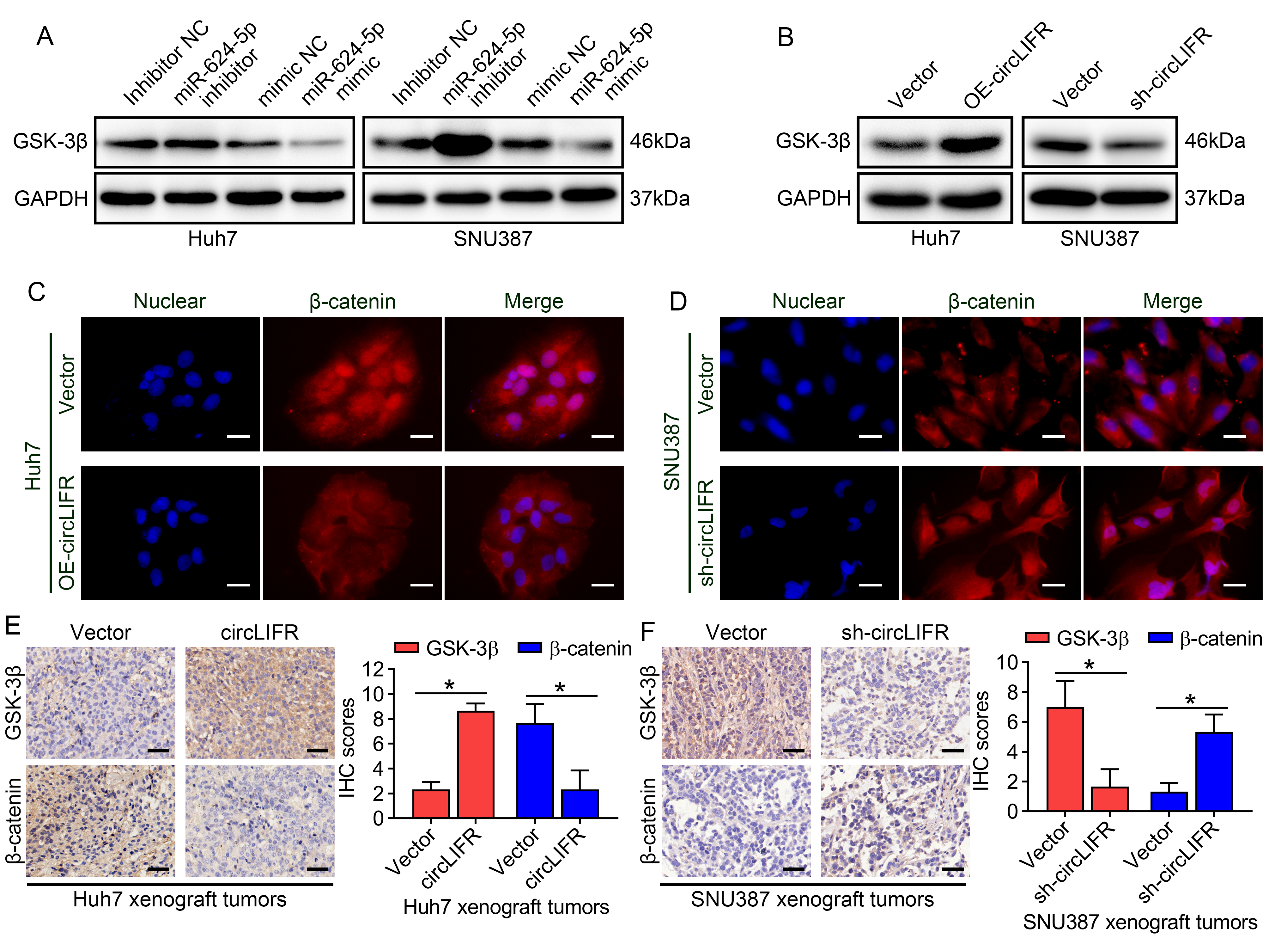

Supplement: Supplementary file 1 — Supplementary Tables and Figures [file 41419_2022_4887_MOESM1_ESM.docx]
