# Supplementary material for: CircLIFR suppresses hepatocellular carcinoma progression by sponging miR-624-5p and inactivating the GSK-3β/β-catenin signaling pathway
Source: Cell Death Dis. 2022 May 17;13(5):464. doi: 10.1038/s41419-022-04887-6 (PMC9114368; doi:10.1038/s41419-022-04887-6)
Supplement: Supplementary file 2 — Supplementary materials [file 41419_2022_4887_MOESM2_ESM.docx]

# Supplementary materials and methods

## The Cell Counting Kit-8 (CCK-8) proliferation assay

The proliferation of Huh7 and SNU387 cells was evaluated with the CCK-8 kit (APExBIO, USA) in accordance with the manufacturer's specifications. Briefly, 2 × 10^3^/well of Huh7 and SNU387 cells were seeded in 96-well plates. Subsequently, 10% CCK-8 reagent was added to each well at five-time points (0, 24, 48, 72, 96 and 120 h), followed by incubation at 37℃ for 2 h. The optional density was measured at 450 nm using an automated ELISA plate reader.

## Colony formation assay

Briefly, 1 × 10^3^ HCC cells were plated in six-well plates and cultured for 2 weeks. Then, cells were washed twice with phosphate-buffered saline (PBS), fixed with 4% paraformaldehyde for 15 min, and then stained with crystal violet staining solution (Beyotime, Shanghai, China) for 10 min. The visible colonies were then photographed and counted.

## Transwell migration and invasion assay

Transwell chambers (Corning, USA) were pre-coated with or without Matrigel basement membrane matrix (Corning, USA). HCC cells were seeded in the upper chambers, loaded with 200 μL of serum-free medium, and plated on 24-well plates. The lower chamber was loaded with 600 μL of medium containing 10% FBS. After 24 or 48 h of incubation, the upper chambers were fixed and stained with crystal violet staining solution (Beyotime, China) for 10 min. Photographs were obtained using a Nikon Eclipse 80i microscope (Japan).

## RNA extraction and quantitative real-time polymerase reaction (qRT-PCR)

Total RNA from HCC cells and tissues was isolated with a RNA purification kit (EZBioscience, Roseville, MN, USA), and gDNA from HCC cells was extracted with a genomic DNA extraction kit (TIANGEN, Beijing, China), according to the manufacturer's instructions. According to the manufacturer's protocol, cytoplasmic and nuclear fractions of cells were isolated using the PARIS kit (Life Technologies, USA). The concentration and purity of RNA samples were measured using Nanodrop One (Thermo Scientific, USA). Total RNA was converted to cDNA using Evo M-MLV RT premix (Accurate Biotechnology, Hunan, China) following the manufacturer's instructions.

qRT-PCR assays were performed using a 2 × SYBR Green Pro Taq HS qPCR kit (AGbio, Hunan, China) on a Bio-Rad CFX Connect system (Bio-Rad, USA). GAPDH and U6 were used as internal controls, and each sample was performed in triplicate. The results of the analysis are expressed as fold change (2^-ΔΔCT^). The back splice site of circLIFR was detected by divergent primers, while convergent primers were used to detect linear LIFR mRNA. The relative amounts of U6, GAPDH and circLIFR in the cytoplasm and nucleus were detected by qRT-PCR. U6 was used as a nuclear control and GAPDH as a cytoplasmic control. Primer sequences are listed in supplementary Table 4.

## Dual-luciferase reporter assay

The 3'UTRs of circLIFR, GSK-3β and their corresponding mutant sequences were inserted into the dual-luciferase reporter pmiR-RB-REPORTTM (RiboBio, China). These plasmids are referred to as circLIFR-WT, circLIFR-MUT, GSK-3β-3'UTR-WT and GSK-3β-3'UTR-MUT. Huh7 cells were transfected with luciferase reporter plasmids, and miR-624-5p mimic or mimic NC. After 24 h of incubation, relative luciferase activity was checked with the Dual-Luciferase Assay Kit (Promega, USA) according to the manufacturer's protocol.

## Western blot

Cells were harvested and lysed in RIPA lysis buffer (CWBio, China) containing protease inhibitors (CWBio, China) for 30 min. Protein concentrations were quantified by bicinchoninic acid analysis (CWBio, China). Protein samples were then separated by 10% SDS-PAGE gels (EpiZyme, Shanghai) and transferred to polyvinylidene fluoride membrane (PVDF, Millipore, USA). After blocking in protein-free fast blocking buffer (EpiZyme, China) for 30 min, the primary antibody was incubated with anti-GAPDH (#AC002) (ABclonal, Wuhan, China), anti-β-catenin （51067-2-AP） (Proteintech, MA, USA) and anti-GSK-3β （22104-1-AP）(Proteintech) overnight at 4℃. Next, incubation with HRP-labeled goat anti-mouse/rabbit IgG (EpiZyme, China) was performed for 1 h. Finally, the bands were visualized with the ECL chemiluminescence detection kit (Millipore, Germany).

## Immunohistochemistry (IHC) testing

HCC tissue samples were fixed in 4% paraformaldehyde, embedded in paraffin, and sectioned. Sections were then dewaxed in xylene and rehydrated in different grades of ethanol. After antigen retrieval in ethylenediaminetetraaceticacid, sections were incubated with anti-Ki-67, anti-β-catenin or anti-GSK-3β primary antibody overnight at 4℃ and then incubated with secondary antibody for 2 h at room temperature. Then, the sections were stained with DAB and hematoxylin. Images were taken with a microscope (Olympus, Japan).

## Immunofluorescence assay

HCC cells were treated for 48 h, and fixed with 4% paraformaldehyde for 20 min. Then, the cells were permeabilized with 0.3% Triton X-100 for 10 min, and blocked with goat serum for 20 min. Next, primary antibody of β-catenin (1:200) was added to the well for incubation overnight at 4°C. After washed with PBS, the cells were incubated with (Alexa Fluor® 555)-labeled secondary antibody for 1h and counterstained with DAPI for 5 min. Finally, the cells were photographed with a fluorescence microscopy.

# Supplementary figure lengends

**Figure S1. Validation and expression of circLIFR in HCC. (**A) Hsa_circ_0072309 was originated from exons 2-5 of its host gene LIFR. Sanger sequencing confirmed the back splice sites of circLIFR which was indicated by the red arrow. (B) Agarose electrophoresis image of PCR amplification with divergent and convergent primers proved the product of circLIFR from complementary DNA (cDNA) or genomic DNA (gDNA) in Huh7 and SUN387 cells.

**Figure S2. The efficiency of knocked down or overexpressed plasmid specific to circLIFR in HCC cells.** (A) Schematic illustration of circLIFR shRNA (sh-circLIFR) #1 and sh-circLIFR#2 targets the back-splice sites of circLIFR. (B) The expression of circLIFR and LIFR mRNA was analyzed by qRT-PCR in Huh7 cells transfected with circLIFR-overexpressing lentivirus or control lentivirus. **(**C**)** The expression of circLIFR and LIFR mRNA was evaluated by qRT-PCR in SNU387 cells transfected with sh-circLIFR plasmid. Data represent means ± SD of three independent experiments. ^*^*P* < 0.05.

**Figure S3. Down-regulation of circLIFR increases HCC progression *in vivo*.**

(A-B) Image of subcutaneous tumors from sh-circLIFR group and control group after injection of SNU387 cells (n=6 for each group). (C) The tumor volume is shown to be notably increased in SNU387 sh-circLIFR group compared with that in control group. (D) The tumor weight is significantly higher in sh-circLIFR group than that in control group. (E) Representative images of xenografts stained with Ki-67 by IHC. Images at 200 × (left panel) and 400 × (right panel) magnification. Scale bar: left =100 μm; right =50 μm. (F) Representative images of orthotopic xenograft tumor growth over time and quantitative analysis of the relative fluorescence intensity over time in SNU387 vector and sh-circLIFR groups (n=3 for each group). (G) Photographs of orthotopic xenograft tumor form SNU387 vector and sh-circLIFR groups and matched xenograft tumor H&E staining are shown. Images were photographed at 100 × (left panel) and 400 × (right panel) magnification. Scale bar: left =200 μm; right =50 μm. Data are represented as mean ±SD；^*^*P* < 0.05.

**Figure S4. CircLIFR binds to miR-624-5p in HCC cells.** (A) RNA immunoprecipitation (RIP) assay was performed to validate circLIFR can sponge miRNAs by using the antibody of Ago2. (B) The co-localization of circLIFR and miR-624-5p was confirmed by FISH assay with CY3-labeled circLIFR probe and FAM labeled miR-624-5p probe. (C) qRT-PCR results to show the expression of miR-624-5p in our cohort of 60 pairs of HCC and adjacent normal tissue. The pictures were 1000 × magnified. Scale bar: 10 μm.

**Figure S5.** **MiR-624-5p inhibits the expression of GSK-3β in HCC cells.** (A) The protein levels of GSK-3β after transfected with miR-624-5p mimics or inhibitor in Huh7 and SNU387 cells. (B) Western blot assay to detect the protein levels of GSK-3β after overexpression of circLIFR in Huh7 cells or knocking down circLIFR in SNU387 cells. (C-D) Immunofluorescence detection demonstrated that overexpressing circLIFR could prevent the nuclear translocation of β-catenin in Huh7 cell, while down-regulation of circLIFR could promote the nuclear translocation of β-catenin in SNU387 cell. (E-F) IHC to detect the expression of GSK-3β and β-catenin in the above Huh7 and SNU387 derived xenograft tumors. The pictures were 100 × magnified. Scale bar: 50 μm.
