# Supplementary figures and images for: CircLIFR suppresses hepatocellular carcinoma progression by sponging miR-624-5p and inactivating the GSK-3β/β-catenin signaling pathway
Source: Cell Death Dis. 2022 May 17;13(5):464. doi: 10.1038/s41419-022-04887-6 (PMC9114368; doi:10.1038/s41419-022-04887-6)

**Fig. 6C**

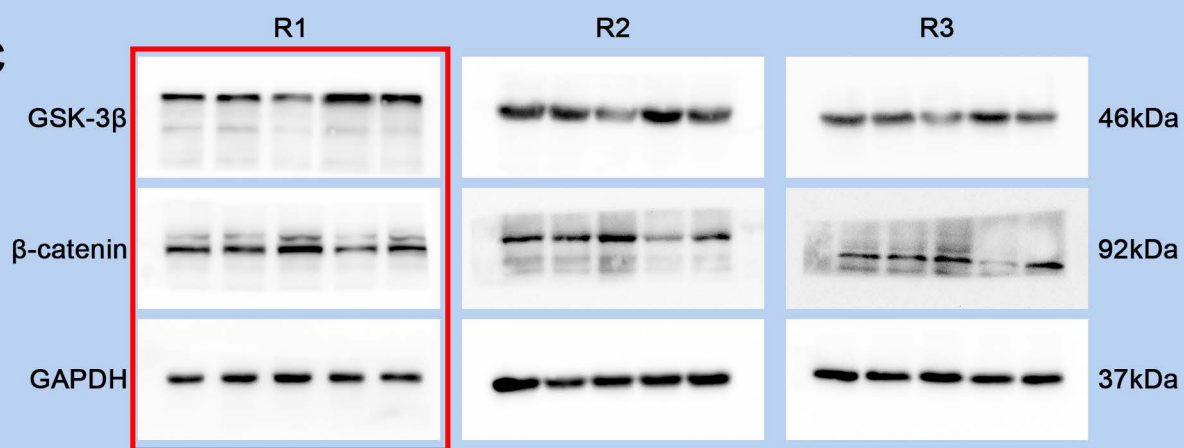

**Fig. 6D**

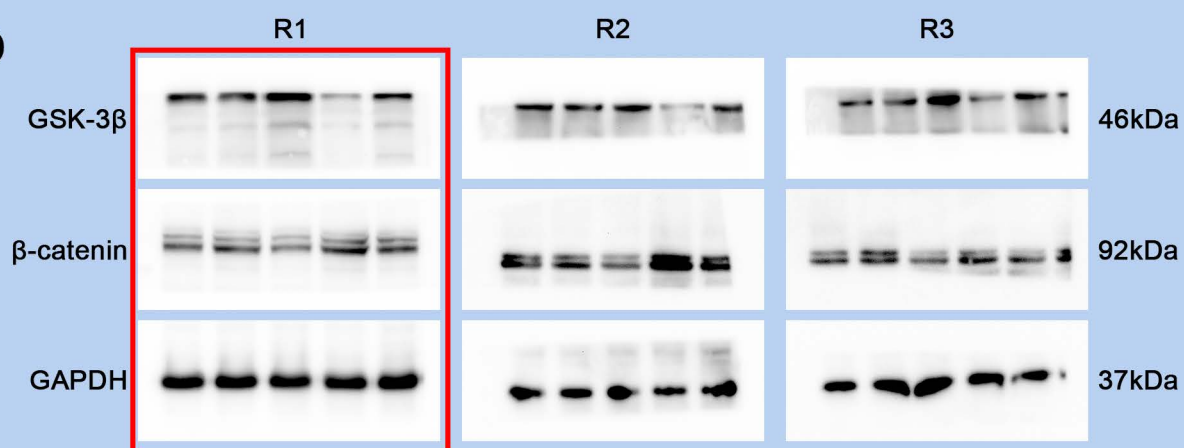

**Fig. 6E**

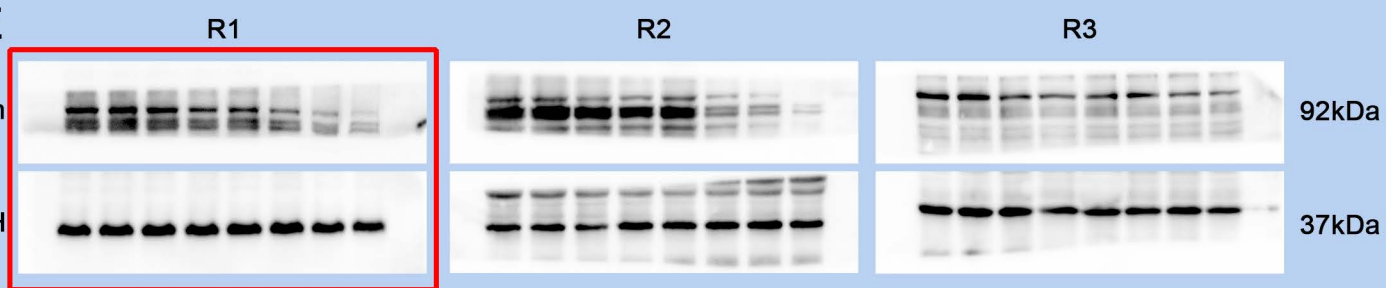

**Fig. 6F**

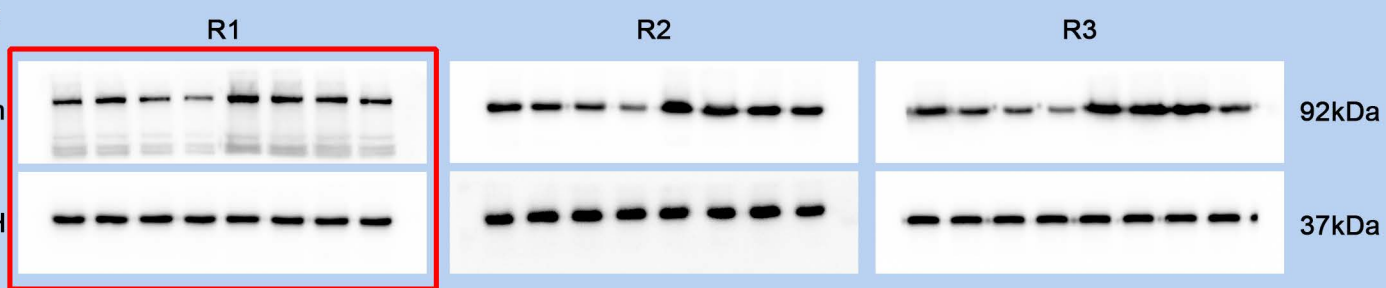

Fig. S5A

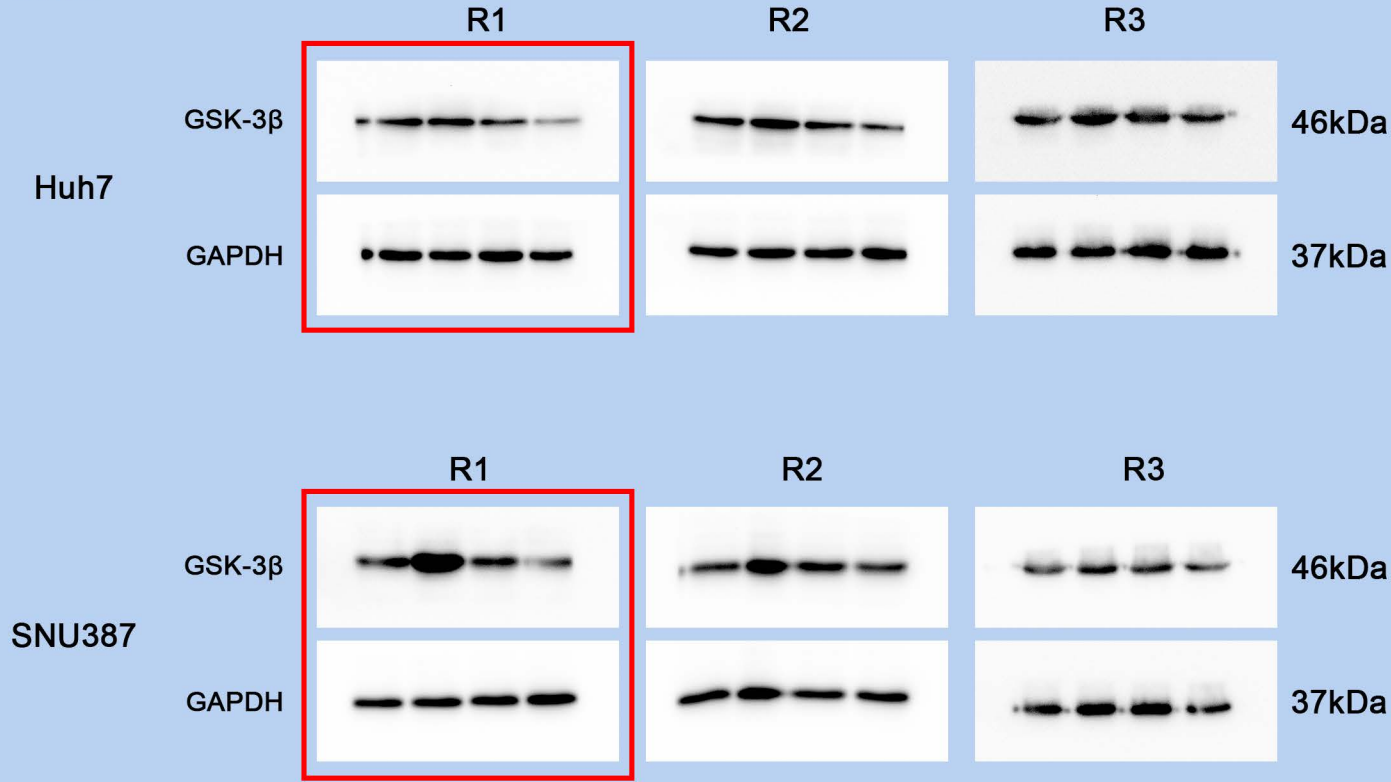

Fig. S5B

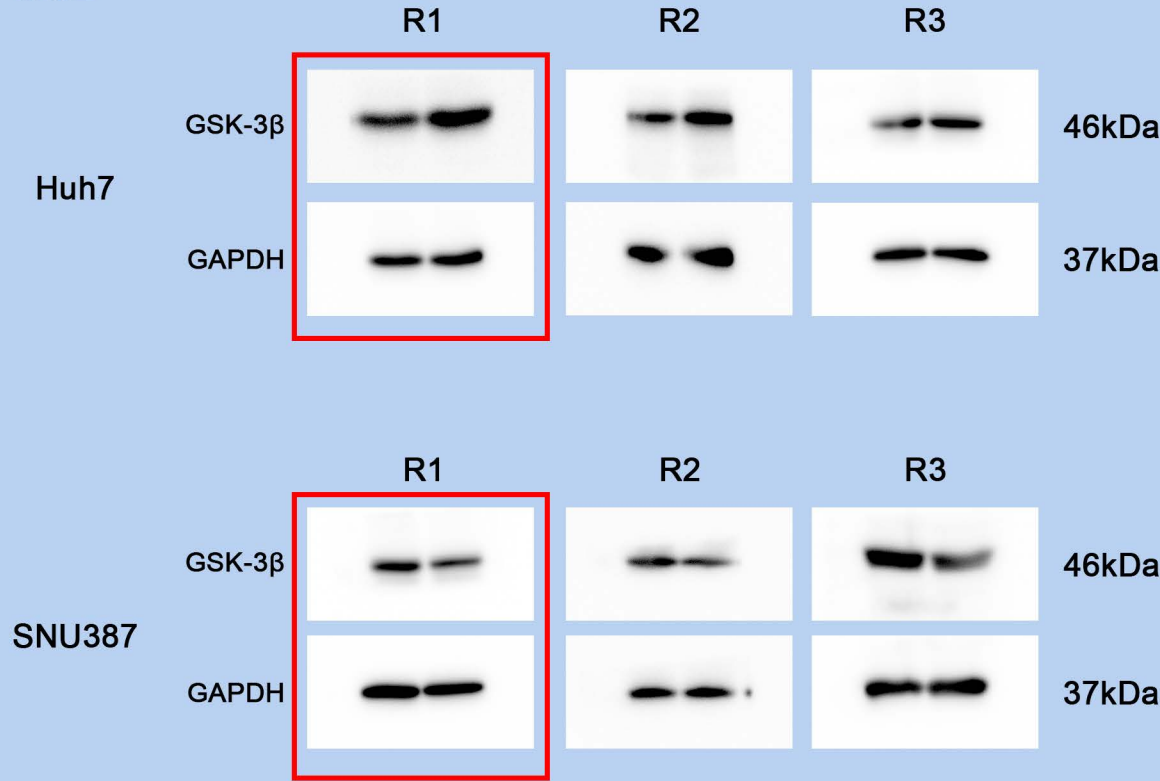

Supplement: Supplementary file 3 — Supplemental Material (Original WB) [file 41419_2022_4887_MOESM3_ESM.pdf]
