# Supplementary material for: CircLIFR suppresses hepatocellular carcinoma progression by sponging miR-624-5p and inactivating the GSK-3β/β-catenin signaling pathway
Source: Cell Death Dis. 2022 May 17;13(5):464. doi: 10.1038/s41419-022-04887-6 (PMC9114368; doi:10.1038/s41419-022-04887-6)
Supplement: Supplementary file 5 — ARRIVE Table [file 41419_2022_4887_MOESM5_ESM.docx]

Animal Research: Reporting In Vivo experiments: The ARRIVE guidelines.

|  | ITEM | | RECOMMENDATION | |
| --- | --- | --- | --- | --- |
| TITLE | | 1 | | CircLIFR suppresses hepatocellular carcinoma progression by sponging miR-624-5p and inactivating the GSK-3β/β-catenin signaling pathway. |
| ABSTRACT | 2 | | | Circular RNAs have been reported to play essential roles in tumorigenesis and progression of various cancers. RNA sequencing data and qRT-PCR results in our cohort identified that circLIFR was markedly down-regulated in HCC tissues. *In vitro* experiments indicated that circLIFR could inhibit the proliferation and invasion abilities of HCC cells,so animal experiments further verified the function of circLIFR. BALB/c male nude mice aged 3-5 weeks were randomly grouped. The construction and observations of subcutaneous and orthotopic xenograft models were performed in a blinded manner. The results of xenograft tumor models showed that over-expressing circLIFR suppressed the proliferation of HCC cells, while inhibition of circLIFR had the opposite effect. |
| INTRODUCTION |  | | |  |
| Background | 3 | | | BALB/c nude mice is a mutant of mice, congenital hairless, no thymus, pure B cell animal. Since nude mice have a congenital T-cell immune deficiency, they can be used as a recipient for xenograft transplantation. BALB/c nude mice can be used to study the monitoring mechanism of carcinogenesis.  The constructed circLIFR overexpression or knockdown stable strain cells were implanted into nude mice, respectively, to observe tumor growth and evaluate the effect of circLIFR on transplanted tumors. |
| Objectives | 4 | | | For subcutaneous xenograft models, the primary objective was the tumor size which were measured weekly with digital calipers. The tumor size was calculated as follows: volume = (width2 × length)/2. For orthotopic xenograft models, the primary objective was bioluminescence images which were examined once a week by Xenogen IVIS Spectrum Imaging System. |
| METHODS |  | | |  |
| Ethical statement | 5 | | | All sample tissues collection was approved by the ethics committee of the Sun Yat-Sen Memorial Hospital of Sun Yat-Sen University. Animal experiments were approved by the Bioethics Committee of Sun Yat-Sen University and were performed according to the National Institutes of Health guidelines, the approval number is SYSU-IACUC-2021-B0113. |
| Study design | 6 | | | In the subcutaneous xenograft models, there were 6 nude mice in the experimental group and the control group respectively. While, in the orthotopic xenograft models, there were 3 nude mice in the experimental group and the control group respectively. In order to minimize the subjective bias, tumor volumes were measured weekly with digital calipers and bioluminescence images were examined once a week both in a blinded manner by one blinded independent investigator. Finally, a single blind method was performed for analysis of results. |
| Experimental procedures | 7 | | | Male BALB/ c nude mice aged 3 to 5 weeks were randomly divided into 6 mice in each group. To establish a subcutaneous xenograft tumor model, a total of 6 × 10^6^ Huh7 OE-circLIFR, Huh7 vector, SNU387 sh-circLIFR and SNU387 vector cells were randomly injected into the back of BALB/c nude mice. To establish an orthotopic xenograft tumor model, 2 × 10^6^ luciferase-expressing Huh7 OE-circLIFR, Huh7 vector, SNU387 sh-circLIFR and SNU387 vector cells were respectively collected into pre-cooled PBS (Thermo Scientific，USA) mixed with matrigel (Corning,NY,USA)in a ratio of 2:1 to form a 50ul system. The mice were anaesthesia by intraperitoneal injection of 4% chloral hydrate (0.1ml /kg) (Sigma-Aldrich USA). The right decubitus position was taken for disinfection by iodophor. An oblique incision was made under the right coal margin with a length of about 1cm. Then,50ul luciferase-expressing cell mixture injected into the right liver parenchyma of mice with a 1-ml insulin syringe. Electrocoagulation to stop bleeding. The abdominal cavity was closed, the wound was sutured and covered with gauze. |
| Experimental animals | 8 | | | The experimental animals were male BALB/c nude mice (bodyweight 10–14g) aged 3-5 weeks. These BALB/c nude mice were purchased from the Laboratory Animal Center of Sun Yat-sen University east Campus. BALB/c nude mice is a mutant of mice, congenital hairless, no thymus, pure B cell animal. Since nude mice have a congenital T-cell immune deficiency, they can be used as a recipient for xenograft or allograft tissue transplantation. BALB/c nude mice, as recipients of human malignant tumor transplantation, can be used to study the monitoring mechanism of carcinogenesis and immunology, the invasion and metastasis mechanism of malignant tumor, the screening of anticancer drugs and the carcinogenicity of cultured cells in vitro. |
| **Housing and husbandry** | 9 | | | BALB/ c nude mice were reared in a SPF barrier system at the Laboratory Animal Center, North Campus of Sun Yat-sen University. The temperature of the feeding environment is 18~29℃.Feed should be aseptic and nutritious, and feed for 3-4 days at a time. Lighting conditions are l2 hours bright, L2 hours dark. The padding material in each cage should be autoclaved before use and the water flows from the filter to clean water, which is changed twice a week. Five to six mice were housed per cage. |
| Sample size | 10 | | | To establish a subcutaneous xenograft tumor model, twenty-four male BALB/ c nude mice aged 3 to 5 weeks were randomly divided into 4 groups with 6 mice in each group. The selected sample size for each group is based on the premise that significant differences can be ensured. To establish an orthotopic xenograft tumor model, twelve male BALB/ c nude mice aged 3 to 5 weeks were randomly divided into 4 groups with 3 mice in each group. The selected sample size was based on feasibility and cost. |
| Allocating animals to experimental groups | 11 | | | Twenty-four male BALB/ c nude mice aged 3 to 5 weeks were randomized blindly divided into 4 groups with 6 mice in each group, and then randomly divided into experimental group and control group. Tumor volumes and bioluminescence images were measured weekly in a blinded manner. |
| Experimental outcomes | 12 | | | For subcutaneous xenograft models, the primary experimental outcomes are to measure tumor size. For orthotopic xenograft tumor model, the primary experimental outcomes are the bioluminescence images. |
| Statistical methods | 13 | | | Statistical analysis (presented as mean ± standard deviation) was performed with GraphPad Prism version 8.0 using Student's t-test. |
| RESULTS |  | | |  |
| Baseline data | 14 | | | Due to BALB/ c nude mice aged 3 to 5 weeks were randomized blindly, the baseline was consistent within each group. |
| Numbers analysed | 15 | | | The number of animals in each group is described above |
| Outcomes and estimation | 16 | | | The results presented as mean ± standard deviation. |
| Adverse events | 17 | | | These is no important adverse events in each experimental group. |
| DISCUSSION |  | | |  |
| Interpretation/scientific implications | 18 | | | The tumor weight and tumor volume in the circLIFR overexpression group were much lower than control group, while circLIFR shRNA had the opposite effect. The results of orthotopic xenograft tumor models showed that overexpression of circLIFR had lower fluorescence intensity in the liver region, while inhibition of circLIFR had the opposite effect.These results showed that circLIFR inhibit the proliferation of liver cancer . |
| Generalisability/translation | 19 | | | Since BALB/c nude mice have a congenital T-cell immune deficiency, they can be used as a recipient for xenograft or allograft tissue transplantation. BALB/c nude mice, as recipients of human malignant tumor transplantation, can be used to study the monitoring mechanism of carcinogenesis and the invasion and metastasis mechanism of malignant tumor.So the findings of this study are likely to translate to human biology. |
| Funding | 20 | | | This work was supported by grants from the National Natural Science Foundation of China (No. 81972263, 82072714 and 82103221), the program of Guangdong Provincial Clinical Research Center for Digestive Diseases (2020B1111170004) and China Postdoctoral Science Foundation (2020M683094). |
|  |  | | |  |
